# Single-cell atlas reveals characteristic changes in intrahepatic HBV-specific leukocytes

**DOI:** 10.1128/spectrum.02860-23

**Published:** 2023-11-30

**Authors:** Banglun Pan, Zengbin Wang, Rui Chen, Xiaoxia Zhang, Jiacheng Qiu, Xiaoxuan Wu, Yuxin Yao, Yue Luo, Xiaoqian Wang, Nanhong Tang

**Affiliations:** 1 Department of Hepatobiliary Surgery, Fujian Institute of Hepatobiliary Surgery, Fujian Medical University Union Hospital, Fuzhou, China; 2 Department of Immunology, School of Basic Medical Sciences, Fujian Medical University, Fuzhou, China; 3 Cancer Center of Fujian Medical University, Fujian Medical University Union Hospital, Fuzhou, China; 4 Key Laboratory of Ministry of Education for Gastrointestinal Cancer, Fujian Medical University, Fuzhou, China; Oklahoma State University College of Veterinary Medicine, Stillwater, Oklahoma, USA

**Keywords:** mass cytometry, HBV, intrahepatic leukocytes, detailed leukocyte ratio

## Abstract

In the progression of liver disease, various metabolic disorders and inflammatory factors cause dysfunction of intrahepatic leukocytes, but there is no research to summarize the changes in the liver microenvironment driven by different pathological factors, especially HBV. Therefore, this study attempted to explore the inherent laws of disease-driven microenvironment changes in diseased livers. Using mass cytometry, flow cytometry, and immunofluorescence, we analyzed the changes in intrahepatic leukocytes from mouse liver diseases and patients with hepatocellular carcinoma (HCC) in this study. We noticed that (i) the inhibitory effect of hepatitis B virus (HBV) on intrahepatic leukocytes did not increase with the surface antigen titers compared with the low hepatitis B surface antigen concentration group, intrahepatic T cells from the high one showed higher expression of PD-1 and lower expression of TIM-3 and CTLA-4, so the proportion of exhausted T cells decreased in the high one, which contributed to the tolerance or adaptation of the liver to HBV; (ii) leukocytes in HBV-associated HCC were in a highly dysfunctional state with highest PD-1/TIM-3/CTLA-4/TOX; (iii) the detailed leukocyte ratio was appropriate to assess the prognosis of HCC, the ratio of cytotoxic/naive/effector/central memory CD8^+^ T cells to CD8^+^ T cells was positively correlated with the overall survival. In conclusion, our phenotype analysis deeply revealed the function differences in intrahepatic leukocytes caused by different liver disease factors, highlighting their unique changes in HBV related HCC, and helping to discover new HCC targeted immunotherapy strategies.

**IMPORTANCE:**

Hepatitis B virus (HBV)-specific CD8^+^ T cells play a central role in the clearance of virus and HBV-related liver injury. Acute infection with HBV induces a vigorous, multifunctional CD8^+^ T cell response, whereas chronic one exhibits a weaker response. Our study elucidated HBV-specific T cell responses in terms of viral abundance rather than the timing of infection. We showed that in the premalignant stage, the degree of impaired T cell function was not synchronized with the viral surface antigen, which was attributed the liver’s tolerance to the virus. However, after the development of hepatocellular carcinoma, T cell exhaustion was inevitable, and it was marked by the exhaustion of the signature transcription factor TOX.

## INTRODUCTION

Heterogeneous leukocyte subsets in the complex hepatic microenvironment are involved in maintaining immune homeostasis. During the progression from liver injury, cirrhosis to hepatocellular carcinoma (HCC), the functions of intrahepatic leukocytes become dysfunctional. When pathological changes occur in the liver, the inherent leukocytes lose their antigen-presenting ability, and cytokines secreted by CD4^+^ T cells are also reduced (1). Macrophages differentiate into an “alternately activated phenotype” and recruit various suppressor leukocytes (2). At the stage of hepatocarcinogenesis, the cytolytic activity of natural killer (NK) cells is inhibited, the functions of T cells are weakened, and the expression of inhibitory receptors is upregulated (1).

In addition, both viral infection and chronic inflammation increase the risk of cancer. Hepatitis B virus (HBV), as one of the most common chronic viral infections, is accompanied by immune damage of the liver, and patients have insidiously progressed to cirrhosis or HCC (3). In chronic HBV infection, T cell exhaustion is one of the important mechanisms of T cell dysfunction (4). But is exhaustion a major barrier mechanism for HBV-specific T cells? The response rate of HBV-associated HCC to anti PD-1/PD-L1 antibodies is less than 30% (5), indicating that blocking the PD-1/PD-L1 pathway is not effective in restoring T cell function. T cell function in the peripheral blood of HBV-infected patients does not show a negative correlation with serum antigen levels, and T cell function does not recover even if hepatitis B surface antigen (HBsAg) is cleared (6). The dysfunction of CD8^+^ T cells is not significantly correlated with the expression of PD-1, nor does it show a correlation with other inhibitor receptors (7, 8). Therefore, characterizing the unique changes in the phenotype of infiltrating T cells in HBV-associated HCC is important for understanding the exhaustion of HBV-specific T cells, finding reliable immune checkpoint markers, and providing insights into the immunotherapy of HBV-associated HCC.

Moreover, the relationship between the composition of leukocytes and prognosis is still controversial. High abundance of CD3^+^ T cells, cytotoxic CD8^+^ T cells, and CD45RO^+^ memory T cells showed a clear positive correlation with longer disease-free survival (DFS) and overall survival (OS) (9). However, the impact of regulatory T cells (TRegs) on survival has been debated, some studies have shown no effect on prognosis, while others have shown a strong association with suboptimal prognosis in head and neck squamous cell carcinoma and HCC (10 – 12). Liver-resident macrophages have been implicated in tumor growth, but whether this affects patient outcomes remains unclear (2). There are also contradictions in the tumor-regulatory roles of antigen-presenting cells (13). Antigen-presenting cells promote the transformation of resting T cells into TRegs to promote metastasis, while other studies have shown that they induce CD4^+^ T cell-dependent CD8^+^ T cell activation, thereby controlling tumor progression (11, 13). Nevertheless, none of these reports have specifically investigated T cell, NK cell, and macrophage infiltration in relation to the overall immune landscape in HCC (especially HCC with different underlying diseases).

Past studies have focused on understanding changes in single type of leukocytes in specific diseases and have not compared the unique changes in immune infiltration and homeostasis across the various lesions. In order to understand the nature of the diseased livers in more detail, we constructed mouse models of liver diseases, collected the resected patient HCC specimens with different underlying diseases, and comprehensively analyzed the characteristic changes of the microenvironment. Our in-depth phenotypic analysis revealed the fundamental differences in leukocyte frequency and function across diseases, highlighting the unique changes in the intrahepatic leukocytes in HBV-associated HCC.

## RESULTS

### Mass cytometry analysis of unique changes in the intrahepatic leukocytes across different diseased livers

**Fig 1:**
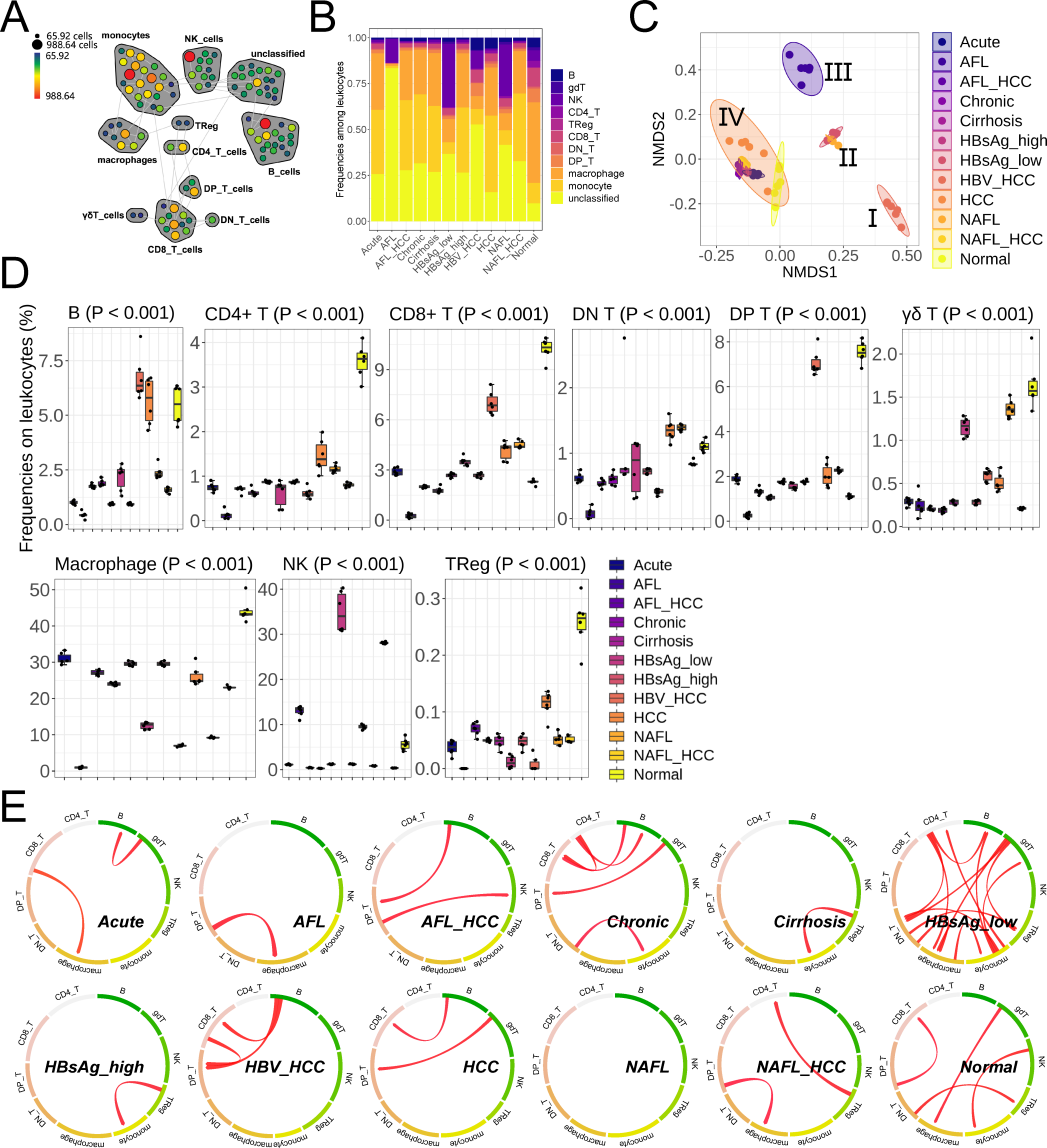
Mass cytometry analysis of unique changes in the intrahepatic leukocytes across different liver models. (**A**) A representative SPADE map showing the meta-clustering of mouse intrahepatic CD45^+^ leukocytes (*n =* 6). (**B**) Composition of the mouse intrahepatic leukocyte subsets (*n =* 6). (**C**) MDS plot showing the mean marker expression across mouse intrahepatic leukocytes (*n =* 6). (**D**) Relative frequency of mouse intrahepatic leukocyte subsets in diseased and normal livers (*n =* 6). (**E**) Circos plots showing the multiple correlation matrix between mouse intrahepatic leukocyte frequencies (*n =* 6). Correlation coefficients higher than 0.9 were represented in red, and lower than −0.9 were represented in blue. Mean ± SD. Statistical significance was evaluated by one-way ANOVA or Kruskal-Wallis test (**D**) and Pearson correlation coefficient (**E**).

The overall strategy involved harvesting freshly resected tissues from the normal liver (Normal) and 11 mouse models of liver diseases (including acute liver injury [Acute], alcoholic fatty liver [AFL], AFL-associated HCC [AFL_HCC], chronic liver injury [Chronic], and cirrhosis [Cirrhosis], HBV infection with low HBsAg concentration [HBsAg_low], HBV infection with high HBsAg concentration [HBsAg_high], HBV-associated HCC [HBV_HCC], HCC without underlying diseases [HCC], non-AFL [NAFL], and NAFL-associated HCC [NAFL_HCC]) and analyzing the composition of the intrahepatic leukocytes by mass cytometry. HBV-associated markers from the HBsAg_low and HBsAg_high groups were detected (Fig. S1), and hepatic tissues were pathologically identified by hematoxylin-eosin (HE) staining (Fig. S2). To visualize each individual leukocyte subset isolated from the different models, we created a two-dimensional map using sequential pattern discovery using equivalence class (SPADE) (Fig. 1A). By reducing the high-dimensional data to two dimensions, we simultaneously quantified the expression of all markers on all cell subsets (Fig. S3). This strategy allowed us to construct a map of different leukocytes, including monocytes/macrophages (CD11b^+^), double negative (DN) T cells (CD3^+^ CD4^-^ CD8^-^), double positive (DP) T cells (CD3^+^ CD4^+^ CD8^+^), CD4^+^ T cells (CD3^+^ CD4^+^), CD8^+^ T cells (CD3^+^ CD8^+^), TRegs (CD3^+^ CD4^+^ CD25^+^ FOXP3^+^), γδ T cells (CD3^+^ TCRgd^+^), B cells (CD19^+^), and NK cells (CD335^+^) (Fig. 1A). It was evident that monocytes/macrophages constituted the majority of the leukocytes, and their proportion was reduced when the liver function was compromised (most pronounced in AFL) (Fig. 1B). We then evaluated the similarity of the models by applying multidimensional scaling (MDS) to various leukocytes, where the mean value of marker expression was used to calculate the distance or relative similarity among samples. The first dimension (I) showed a clear separation between HBV_HCC and other models, the second one (II) pointed out that the relative difference between NAFL and HBsAg_low was not noticeable, the third one (III) implied that AFL was far away from other diseases, and the characteristic of the fourth one (IV) was that Normal/HCC/Acute/Chronic/Cirrhosis/HBsAg_high were close to each other, although there was a clear separation between Normal and five other types of models (Fig. 1C). To better understand the model segregation in the MDS analysis, we visualized the mean marker expression of the leukocytes using a heatmap with unsupervised hierarchical clustering (Fig. S4). The most informative differentially expressed markers were those commonly expressed by macrophages (CD11b, F4/80) and T cells (CD3e).

As shown in Fig. 1D; Fig. S5, the infiltration of CD4^+^/CD8^+^/DP/γδ/regulatory T cells and macrophages was inhibited in all diseased liver tissues. Unlike them, NK cells were significantly increased in AFL/HBsAg_low/HBV_HCC/NAFL (Fig. 1D; Fig. S5). Compared with NAFL, AFL had a stronger inhibitory effect on various leukocytes (Fig. 1D; Fig. S5), and CD45 expression was also significantly inhibited in AFL (Fig. S4). Infiltration of leukocytes was inhibited by both HBsAg_high and HBsAg_low, except NK cells were only inhibited by HBsAg_high, not HBsAg_low (Fig. 1D; Fig. S5).

The relative distribution across leukocytes was closely related to diseases (Fig. 1E). In Acute/AFL/AFL_HCC/Cirrhosis/HBsAg_high/HCC/NAFL/NAFL_HCC, the link among different leukocytes was weak (Fig. 1E). There were complex communications among T cells, B cells, and macrophages in HBsAg_low, but only monocytes and TRegs were related to each other in HBsAg_high (Fig. 1E).

### NK cell activity was driven by liver disease types

**Fig 2:**
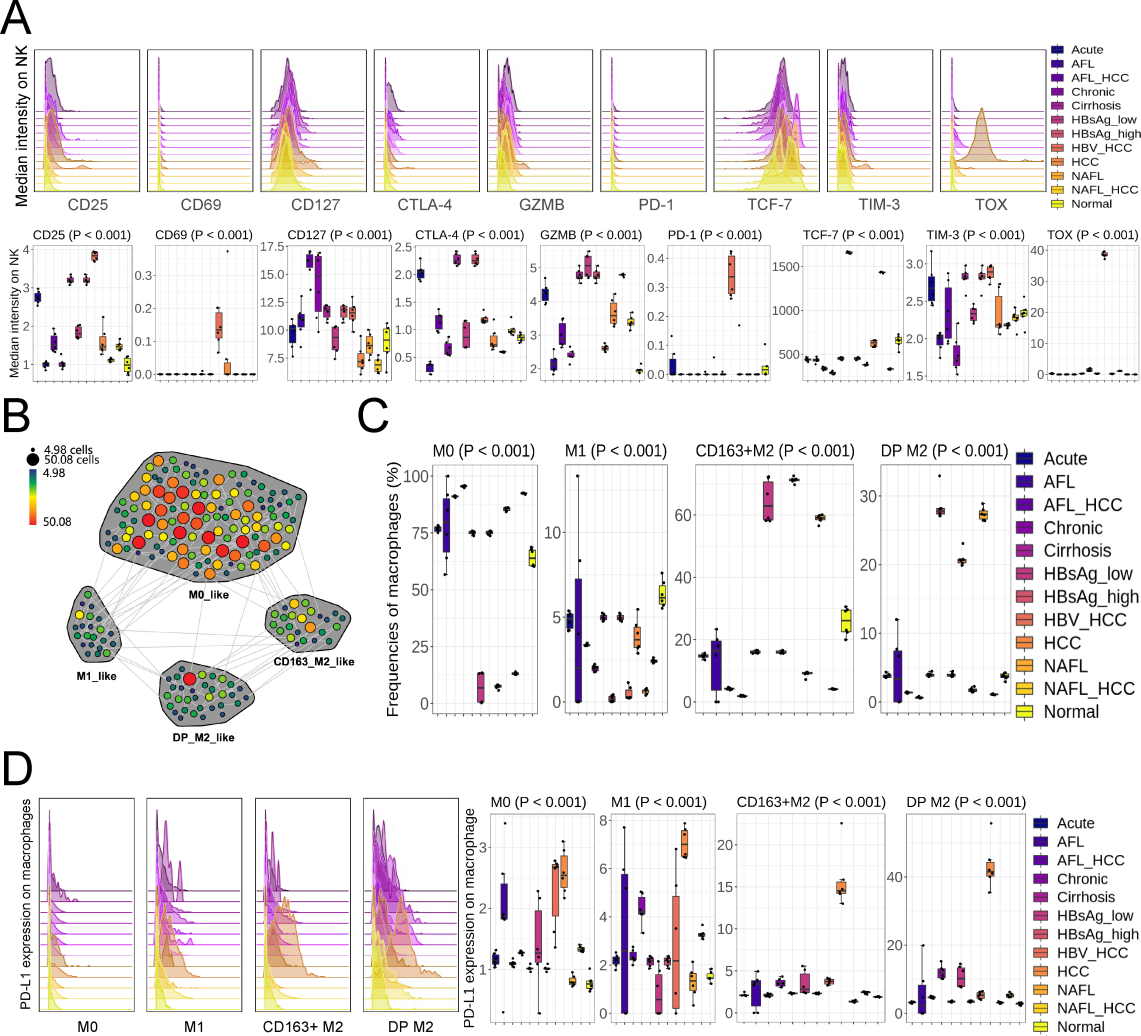
Changes in intrahepatic NK cells and macrophages across different liver models. (**A**) Expression of activation and inhibition genes on mouse intrahepatic NK cells (*n =* 6). (**B**) A representative SPADE map showing the meta-clustering of mouse intrahepatic macrophages (*n =* 6). (**C**) Composition of the mouse intrahepatic macrophage subsets (*n =* 6). (**D**) PD-L1 expression on mouse intrahepatic macrophage subsets (*n =* 6). Mean ± SD. Statistical significance was evaluated by one-way ANOVA or Kruskal-Wallis test (**A, C, and D**).

We examined the expression of the inhibitory and active markers to evaluate NK cell function in the different pathological liver tissues (Fig. 2A). CD25 (activated markers) was induced on NK cells, especially in HBV_HCC (Fig. 2A). TIM-3 and CTLA-4 used to mark inhibitory phenotype were downregulated in AFL/Chronic/NAFL (Fig. 2A). Unlike TIM-3 and CTLA-4, PD-1 and TOX showed strong specificity for HBV_HCC (Fig. 2A), only HBV_HCC was able to induce PD-1 and TOX expression on NK cells (Fig. 2A). NK cells in HBV_HCC were notable, they exhibited the highest active to stemness ratio (highest CD69:TCF-7 or CD25: TCF-7), the most severe exhaustion (highest PD-1/TIM-3/TOX expression), and low cytotoxicity (GZMB expression was inhibited) (Fig. 2A). The above results indicated that the activity and cytotoxicity of NK cells were elevated in liver diseases, but accompanied by different degrees of exhaustion, and HBV_HCC was the most representative disease among them.

### HCC infiltrating macrophages expressed the high levels of PD-L1

To investigate the relative contribution of macrophages in liver diseases, we applied SPADE to CD45, CD11b, F4/80, CD163, CD80, and PD-L1 to compartmentalize macrophages (Fig. 2B). By reducing the high-dimensional data to two dimensions, we simultaneously quantified the expression of markers on macrophage subsets (Fig. S6). This strategy allowed us to construct a map of macrophages, including M0-like (CD80^-^ CD163^-^), M1-like (CD80^+^ CD163^-^), CD163^+^ M2-like (CD80^-^ CD163^+^), and DP M2-like (CD80^+^ CD163^+^) macrophages (Fig. 2B). M0-like macrophages made up the majority of macrophages inAcute/AFL/AFL_HCC/Chronic/Cirrhosis/HBsAg_high/HCC/NAFL_HCC/Normal (Fig. 2B and C). M1-like macrophage infiltration was inhibited upon various liver diseases, and the proportion of CD163^+^ and DP M2-like macrophages was increased in HBsAg_low/HBV_HCC/NAFL (Fig. 2B and C). In addition, we found that PD-L1 on four macrophage subsets was all induced in HCC (Fig. 2D).

### Chronic HBV was characterized by quantitatively and qualitatively weak HBV-specific T cell responses

**Fig 3:**
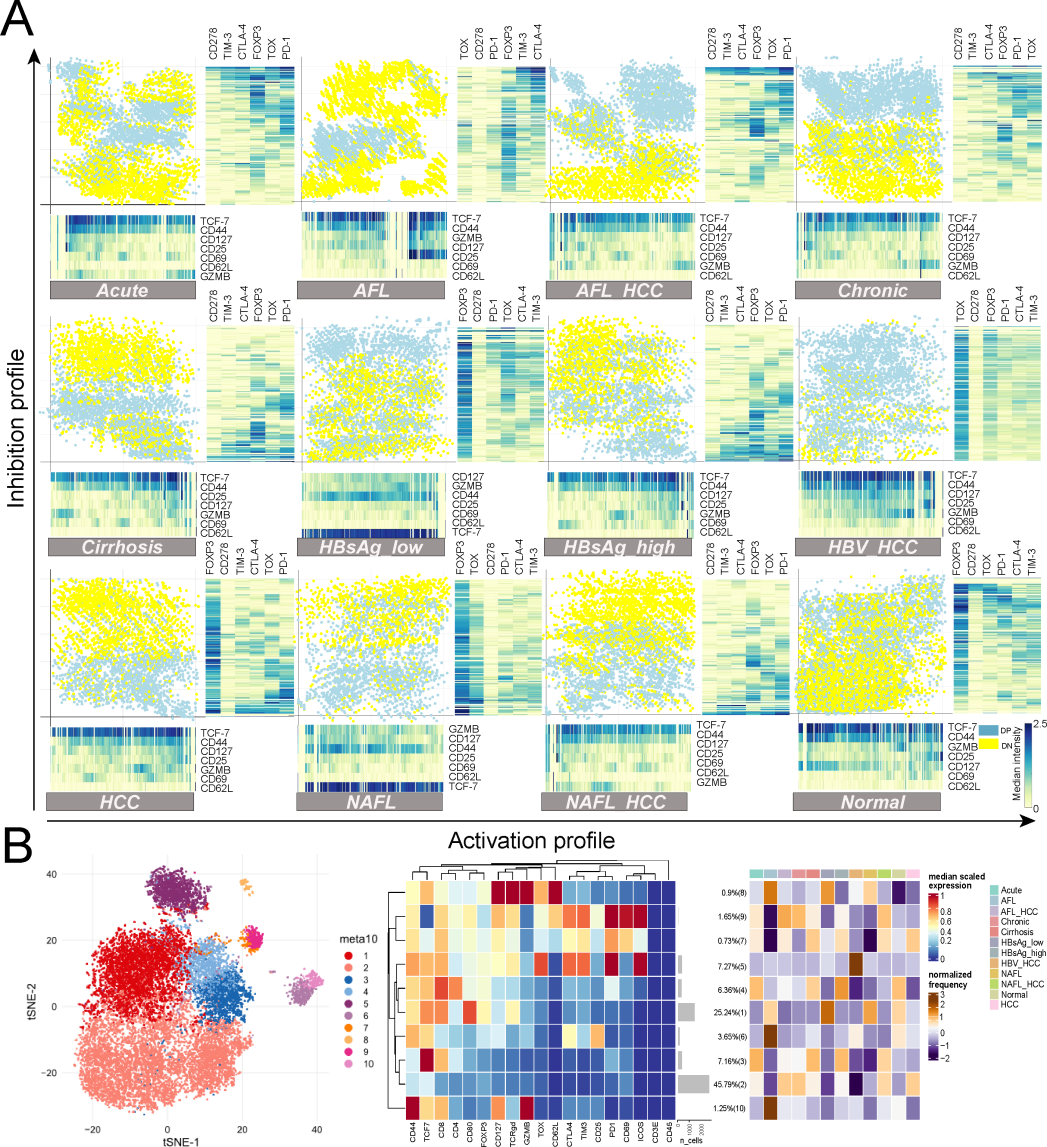
Chronic HBV was characterized by quantitatively and qualitatively weak HBV-specific T cell responses. (**A**) One-SENSE analysis comparing the activation and inhibition profiles of mouse intrahepatic T cells (*n =* 6). (**B**) Left part: a representative t-SNE map showing the FlowSOM-guided meta-clustering of mouse intrahepatic T cells (*n =* 6). Right part: heatmap showing mean expression of T cell markers calculated by T cells among 12 types of mouse liver models (*n =* 6).

We analyzed the phenotype of intrahepatic T cells in more detail with the help of one-dimensional soli-expression by nonlinear stochastic embedding (One-SENSE) analysis (14). The *x*-axis represented the activation profile, and the *y*-axis represented the inhibition profile, including the expression of co-stimulatory and co-inhibitory markers (Fig. 3A). Based on the expression of two inhibitory receptors, PD-1 (a marker of classical exhausted T cells) and TOX (a marker of terminally exhausted T cells), T cells were divided into PD-1^positive^ TOX^positive^ (double positive) and PD-1^negative^ TOX^negative^ (double negative) T cells (Fig. 3A; Fig. S7A). Most of the T cells in Normal were DN T cells (Fig. 3A; Fig. S7B). The relative frequency of DN T cells was decreased and DP T cells were increased in Acute/AFL_HCC/Chronic/Cirrhosis/HBsAg_low/HBsAg_high/HBV_HCC/NAFL/NAFL_HCC/HCC (Fig. 3A; Fig. S7B). Among them, DP T cells in HBV_HCC became the main component of intrahepatic T cells (Fig. 3A; Fig. S7B). Further analysis of T-cell markers by One-SENSE analysis showed that DN T cells in the diseased livers had increased activity (elevated CD69 expression), while DP T cells had decreased activity (decreased CD69 expression) (Fig. 3A; Fig. S7C). Cytotoxicity (GZMB) of DN and DP T cells was increased in HBsAg_low and NAFL but decreased in the other models (Fig. 3A; Fig. S7C). The stemness (TCF-7) of DN and DP T cells was enhanced in HBsAg_low/HCC/NAFL (Fig. 3A; Fig. S7C). The exhaustion phenotype of HBV_HCC infiltrating T cells was distinctive, PD-1, TIM-3, and TOX were induced on both T cell subsets, and the elevation of TOX was the most obvious (Fig. 3A; Fig. S7C).

We performed the FlowSOM algorithm using differentially expressed markers (15) to explore the landscape of intrahepatic T cells (Fig. 3B). Our analysis showed that T cells were divided into 10 subsets, namely Cluster 1 (CD80^high^ CD8^+^ T cells), Cluster 2 (DN T cells), Cluster 3 (TCF-7^high^ CD8^+^ T cells), Cluster 4 (DP T cells), Cluster 5 (TOX^high^ CTLA-4^high^ TIM-3^high^ PD-1^high^ ICOS^high^ CD8^+^ T cells), Cluster 6 (CD25^mid^ DN T cells), Cluster 7 (CTLA-4^mid^ TIM-3^mid^ PD-1^mid^ CD8^+^ T cells), Cluster 8 (γδ T cells), Cluster 9 (CTLA-4^high^ TIM-3^high^ PD-1^high^ ICOS^high^ CD8^+^ T cells), and Cluster 10 (GZMB^high^ CD8^+^ T cells) (Fig. 3B). The effect of disease types on the proportion of T cell subsets was obvious (Fig. 3B). Cluster 3 was elevated in Acute/Cirrhosis/HBsAg_high, Cluster 5 in HBV_HCC, Cluster 7 in Acute/AFL_HCC/Cirrhosis/HBsAg_high/NAFL_HCC/HCC, Cluster 8 in all liver lesions, Cluster 9 in Acute/AFL_HCC/Chronic/NAFL_HCC, and Cluster 10 in AFL (Fig. 3B).

### Impaired liver function was accompanied by incomplete T cell function

**Fig 4:**
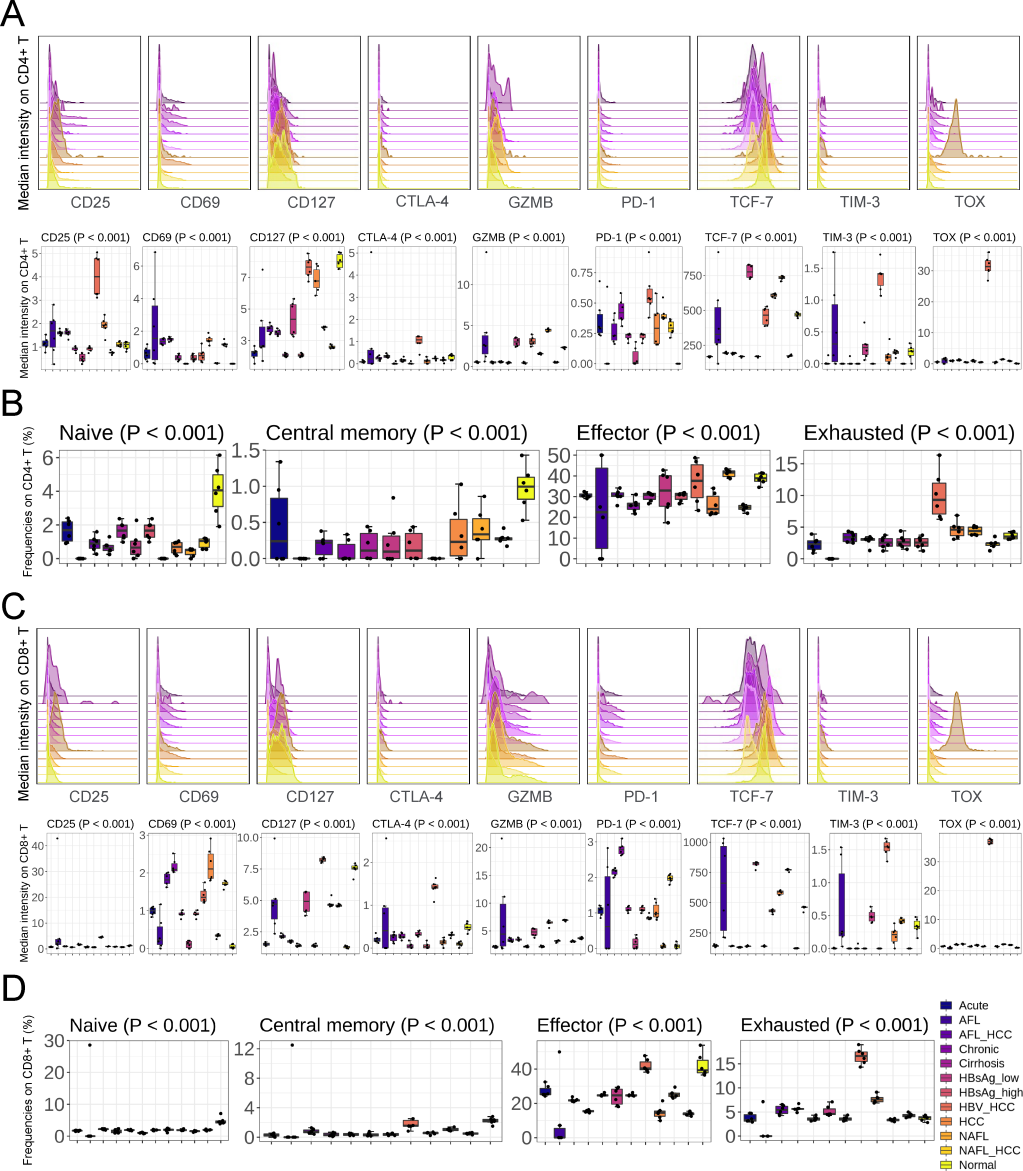
Impaired liver function was accompanied by incomplete T cell function. (**A**) Expression of activation and inhibition genes on mouse intrahepatic CD4^+^ T cells (*n =* 6). (**B**) Composition of mouse intrahepatic CD4^+^ T cell subsets (*n =* 6). (**C**) Expression of activation and inhibition genes on mouse intrahepatic CD8^+^ T cells (*n =* 6). (**D**) Composition of mouse intrahepatic CD8^+^ T cell subsets (*n =* 6). Mean ± SD. Statistical significance was evaluated by one-way ANOVA or Kruskal-Wallis test (**A–D**).

As shown in Fig. 3 and Fig. S7, T cell function was primarily driven by disease types. We next explored the effect of disease types on the function of CD4^+^/CD8^+^ T cells. We noticed that the activity of CD4^+^/CD8^+^ T cells was upregulated (increased CD69 and decreased CD127 expression) when liver function was impaired (Fig. 4A and C). Compared with CTLA-4 and TIM-3, PD-1 more accurately reflected the functional impairment of CD4^+^/CD8^+^ T cells, PD-1 was upregulated once liver function was impaired (Fig. 4A and C). Moreover, PD-1 expression increased with HBV antigen titers (Fig. 4A and C). In Acute/Cirrhosis/HBsAg_high/NAFL_HCC, the expression of CTLA-4 and TIM-3 on CD4^+^/CD8^+^ T cells was inhibited instead (Fig. 4A and C). Moreover, the expression of CTLA-4 and TIM-3 on HBsAg_high infiltrating CD8^+^ T cells was lower than HBsAg_low (Fig. 4A and C). Consistent with Fig. 3B, TOX was a highly sensitive marker for HBV_HCC, and only TOX on HBV_HCC infiltrating CD4^+^/CD8^+^ T cells was induced (Fig. 4A and C). Acute/Cirrhosis/HBsAg_high/HCC/NAFL_HCC suppressed GZMB expression on CD4^+^/CD8^+^ T cells, while HBsAg_low/HBV_HCC/NAFL upregulated GZMB expression (Fig. 4A and C). We found that HBV_HCC was a distinct HCC, and the expression of GZMB on infiltrating CD4^+^/CD8^+^ T cells was increased instead, which was consistent with HBsAg_low (GZMB expression was upregulated in HBsAg_low and downregulated in HBsAg_high) (Fig. 4A and C). Previous studies have confirmed our finding: HBV-specific cytotoxic T cells are significantly upregulated during the acute phase and return to basal levels during remission or chronic infection (6, 8). In addition, TCF-7 expression was driven by disease types, HBsAg_low/HCC/NAFL enhanced TCF-7 expression on CD4^+^/CD8^+^ T cells, whereas Acute/AFL_HCC/Chronic/Cirrhosis/HBsAg_high/NAFL_HCC suppressed it (Fig. 4A and C).

To describe the proportion of CD4^+^/CD8^+^ T cells in response to lesions, we divided CD4^+^/CD8^+^ T cells to naive/central memory/effector/exhausted subsets according to the restriction markers (Fig. S8). The percentage of naive and central memory CD4^+^/CD8^+^ T cells in the diseased liver tissues was reduced to varying degrees (Fig. 4B and D). The effect of different lesions on the effector phenotype of CD4^+^/CD8^+^ T cells was not completely consistent (Fig. 4B and D), Acute/AFL_HCC/Chronic/Cirrhosis/HBsAg_high/HCC/NAFL_HCC were seen to inhibit effector CD4^+^/CD8^+^ T cells, and NAFL inhibited effector CD8^+^ T cells (Fig. 4B and D). Compared with other types of HCC, HBV_HCC did not affect effector CD4^+^/CD8^+^ T cells (Fig. 4B and D). Exhaustion of CD4^+^/CD8^+^ T cells was driven by disease types: (i) Acute/Chronic/Cirrhosis induced PD-1 expression on CD4^+^/CD8^+^ T cells (Fig. 4A and C), but not enough to induce CD4^+^/CD8^+^ T cell exhaustion (Fig. 4B and D); (ii) CD8^+^ T cells in AFL_HCC/NAFL_HCC were observed to be exhausted, but not CD4^+^ T cells (Fig. 4B and D); (iii) the most pronounced exhaustion phenotype was observed in HBV_HCC infiltrating CD4^+^/CD8^+^ T cells with highest PD-1/TIM-3/CTLA-4/TOX (Fig. 4A through D); (iv) HBV induced CD8^+^ T cell exhaustion incoherently, compared with HBsAg_low, HBsAg_high infiltrating CD8^+^ T cells showed higher expression of PD-1 and lower expression of TIM-3 and CTLA-4 (Fig. 4A and C), so the proportion of exhausted CD8^+^ T cell decreased in HBsAg_high (Fig. 4B and D).

### Hepatic tolerance to HBV prevented further exhaustion of intrahepatic T cells

**Fig 5:**
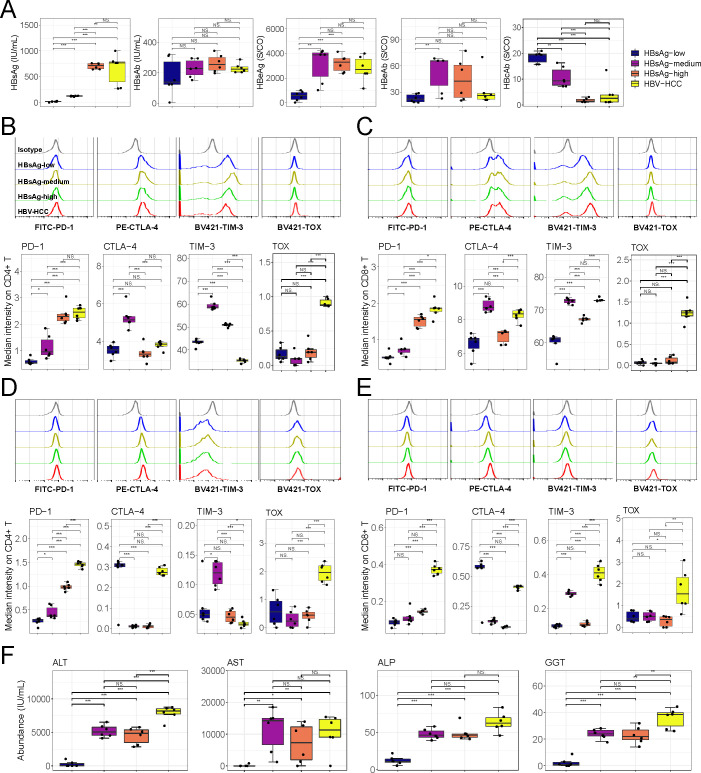
Hepatic tolerance to HBV prevented further exhaustion of intrahepatic T cells in mice. (**A**) The content of HBsAg, HBsAb, HBeAg, HBeAb, and HBcAb in blood (*n =* 6). According to the HBsAg, mice were divided into low, medium, and high groups with cutoff values of 100 and 500 IU/mL, respectively. (**B and C**) Inhibitory gene expression on intrahepatic HBsAg-specific CD4^+^ (**B**) and CD8^+^ (**C**) T cells (*n =* 6). (**D and E**) Expression of inhibitory genes on intrahepatic HBV non-specific CD4^+^ (**D**) and CD8^+^ (**E**) T cells (*n =* 6). (**F**) The content of ALT, AST, ALP, and GGT in blood (*n =* 6). Mean ± SD. Statistical significance was evaluated by Student’s *t* test (**A–F**). *P < 0.05, **P < 0.01, and ***P < 0.001.

Considering that HBV-Tg mice are transgenic mice, not natural HBV-infected, we constructed the mouse model infected with rAAV8-1.3 HBV and compared the difference between HBV-specific and non-specific lymphocytes, so as to accurately characterize the disruption of HBV on intrahepatic lymphocyte function. According to the HBsAg content in blood, we divided the mice into low, medium, and high HBsAg (HBsAg_low, HBsAg_medium, and HBsAg_high) groups and HBV-HCC (Fig. 5A). It has been known that HBV-derived T cell epitopes, including known and putative HBV epitopes of viral proteins, are employed to identify HBV-specific T cells. These antigens include core (HBV-C), polymerase (HBV-P), surface (HBV-S), and x (HBV-X) (8, 16, 17). Therefore, we used surface antigen as a marker of HBV-specific T cells (Fig. S9), and the expression of PD-1, CTLA-4, and TIM-3 on HBV-specific cells was significantly higher than those on HBV non-specific cells, which indicated that HBV induced the exhaustion of T cells (Fig. S10). It could be seen that (i) in HBV-specific T cells, the expression of PD-1 was induced with the increase of HBsAg content; (ii) the expression of CTLA-4 and TIM-3 was upregulated in HBsAg_medium group, but lower than that in HBV-high group; (iii) TOX showed high specificity to HBV-associated HCC (Fig. 5B and C). PD-1 and TOX were seen in HBV non-specific T cells to exhibit a similar expression to HBV-specific cells, but CTLA-4 and TIM-3 did not respond sensitively to HBV (Fig. 5D and E). Two HBV models jointly pointed out that although HBV induced the exhaustion of intrahepatic T cells, which was most severe when progressing to HCC, the expression of CTLA-4 and TIM-3 was not completely synchronized with the content of HBsAg (Fig. 4 and 5). Sakuma et al. have pointed out that asymptomatic carriers carry a high risk of dying from chronic liver disease, and routine liver function tests appear to have limited value in predicting the prognosis (18). Le Bert found that the duration of HBsAg exposure, rather than the quantity of HBsAg, was associated with the level of anti-HBV immune response (19). Therefore, we believed that there was no necessary relationship between the content of HBsAg and liver function. We hypothesized that liver function did not deteriorate with the elevation of surface antigen and that the elevation of antigen stimulated a stronger antiviral immune response, so that T cells in the high titer group showed more toxicity rather than exhaustion than in the low one. We detected alanine aminotransferase (ALT), aspartate aminotransferase (AST), alkaline phosphatase (ALP), and γ-glutamyltransferase (GGT) contents in blood to determine the homeostasis of the liver. We found that compared with HBsAg_low group, the contents of ALT, AST, ALP, and GGT in HBsAg_medium and HBsAg_high groups were significantly increased, but there was no difference between the latter two (Fig. 5F). When developing into HCC, the contents of the four liver function-related indexes reached the peak (Fig. 5F). Therefore, we proposed that HBV induced the exhaustion of intrahepatic T cells, but hepatic tolerance to HBV inhibited the expression of CTLA-4 and TIM-3. Moreover, since PD-1 expression was induced by HBV, we further analyzed the regulatory effect of anti-PD-1 antibody on HBV-associated HCC. We found that the anti-PD-1 antibody alleviated the exhaustion of infiltrating T cells (Fig. S11A), although it did not suppress the content of HBV-associated markers (Fig. S11B).

**Fig 6:**
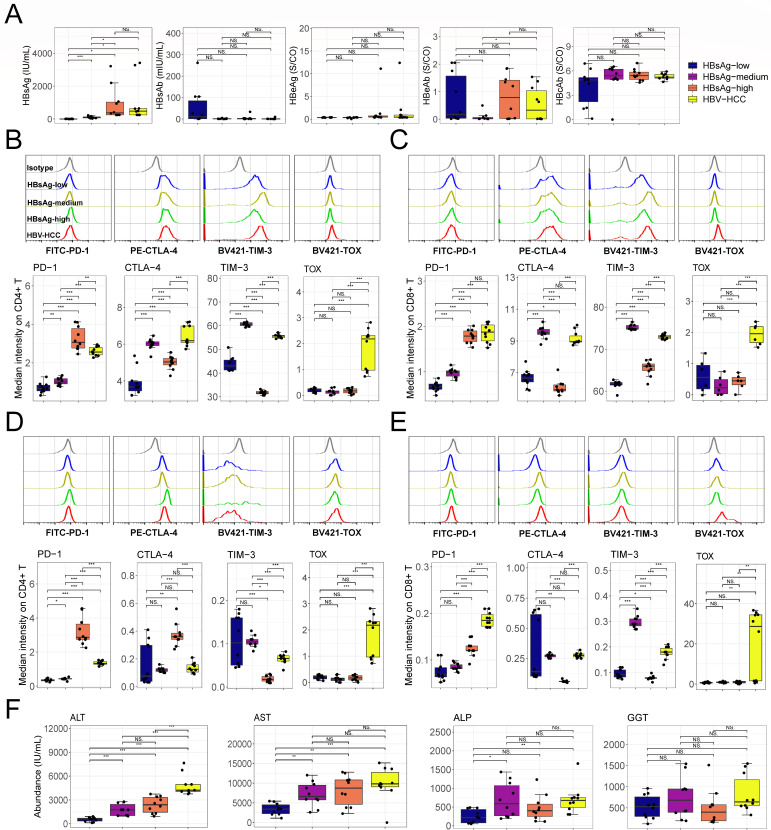
Hepatic tolerance to HBV prevented further exhaustion of intrahepatic T cells in patients. (**A**) The content of HBsAg, HBsAb, HBeAg, HBeAb, and HBcAb in blood (*n =* 10). According to the HBsAg in the blood of patients, they were divided into low, medium, and high groups with cutoff values of 25 and 250 IU/mL, respectively. (**B and C**) Inhibitory gene expression on intrahepatic HBsAg-specific CD4^+^ (**B**) and CD8^+^ (**C**) T cells (*n =* 10). (**D and E**) Expression of inhibitory genes on intrahepatic HBV non-specific CD4^+^ (**D**) and CD8^+^ (**E**) T cells (*n =* 10). (**F**) The content of ALT, AST, ALP, and GGT in blood (*n =* 10). Mean ± SD. Statistical significance was evaluated by Student’s *t* test (**A–F**). *P < 0.05, **P < 0.01, and ***P < 0.001.

In order to judge whether the relationship between HBV and T cell exhaustion derived from the mice was consistent with that of patients, we collected normal (para-cancerous) and tumor tissues from HCC patients with chronic HBV infection and divided them according to the content of HBsAg into four groups (HBsAg_low, HBsAg_medium, HBsAg_high, and HBV-HCC) (Fig. 6A). Undoubtedly, HBV-specific T cells did express higher levels of PD-1, CTLA-4, and TIM-3 compared with HBV non-specific ones (Fig. S10). Moreover, we found that, similar to the mouse HBV infection model (Fig. 5B through E), both in HBV-specific (Fig. 6B and C; Fig. S9) and HBV non-specific (Fig. 6D and E; Fig. S9) T cells, PD-1 expression was induced by HBsAg and TOX was only elevated in HBV-HCC, while CTLA-4 and TIM-3 expression was induced in HBsAg_medium but repressed by HBsAg_high and was strongly upregulated in HBV-HCC. In addition, ALT, AST, and ALP were increased in HBV and HBV-HCC groups, but they were not different between the medium and high HBsAg groups (Fig. 6F).

### Underlying diseases rather than the pathological classification affected T cell function in HCC

**Fig 7:**
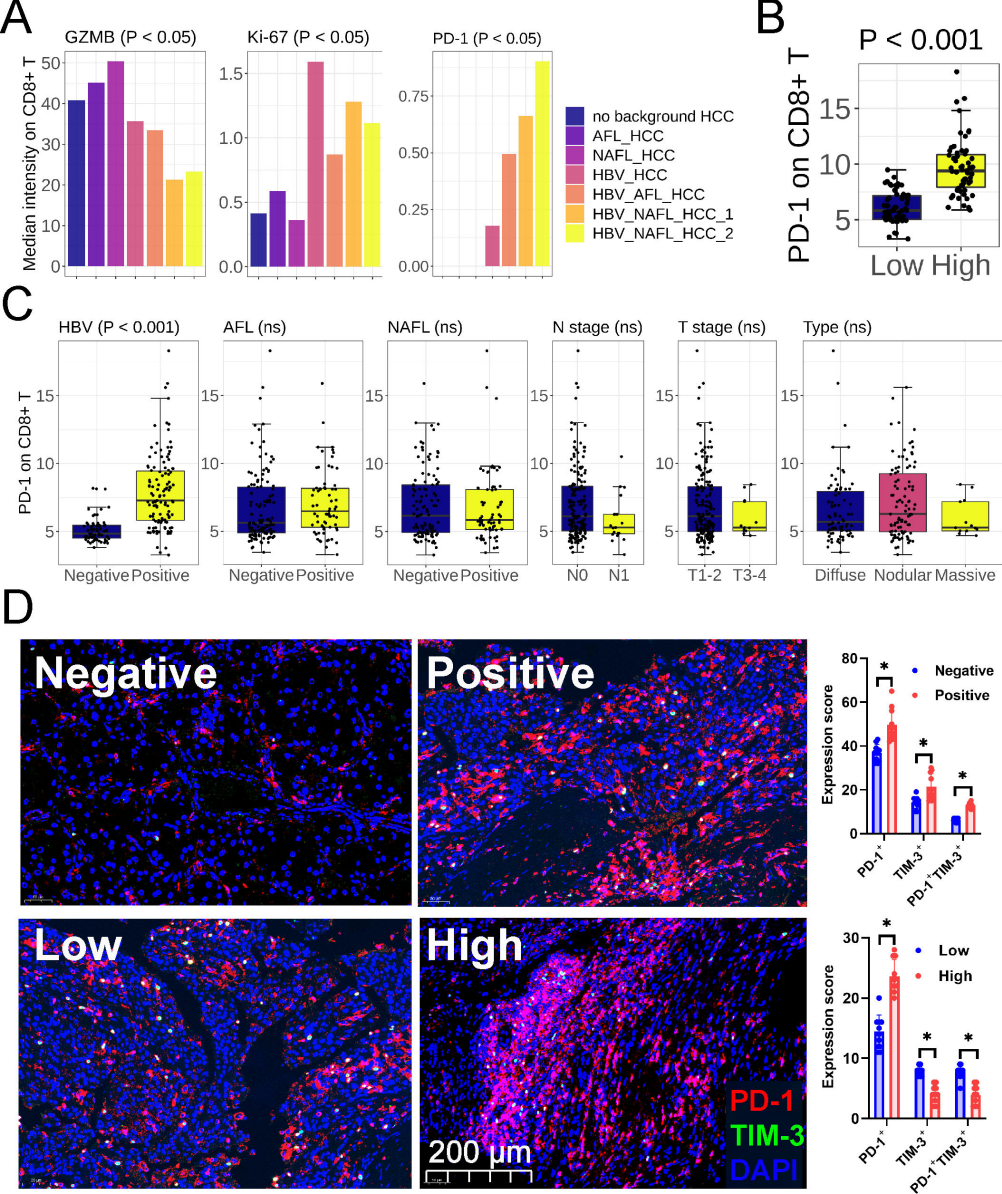
Underlying diseases rather than the pathological classification affected T cell function in HCC. (**A**) Expression of activation and inhibition genes on patient HCC infiltrating CD8^+^ T cells (*n =* 7). (**B and C**) Effect of HBsAg concentration (**B**) and the underlying diseases (**C**) on PD-1 expression on patient HCC infiltrating CD8^+^ T cells (*n =* 189). (**D**) Immunofluorescence showed the expression of PD-1 and TIM-3 in pathological sections of the patients with HCC (*n =* 10). Negative, HBV infection negative; positive, HBV infection positive; low, low HBsAg concentration (<127 IU/mL); high, high HBsAg concentration (≥127 IU/mL). Mean ± SD. Statistical significance was evaluated by Student’s *t* test (**A–D**) and Kruskal-Wallis test (**C**).

Since in the mice with HBV-associated HCC, infiltrating T cells exhibited the most exhaustion phenotype (Fig. 3 to 6), we next validated this finding in patients’ HCC infiltrating T cells. The overall strategy consisted of collecting fresh leukocytes from HBV^negative^ HCC (AFL_HCC, NAFL_HCC, no background HCC) and HBV^positive^ HCC (HBV_HCC, AFL + HBV_HCC, NAFL + HBV_HCC) and analyzing the composition of leukocyte subsets. The effect of HBV on the proportion of infiltrating leukocytes was minimal (Table S1). Compared with HBV^negative^ HCC, infiltrating CD8^+^ T cells in HBV^positive^ HCC had higher expression of proliferation marker (Ki-67) and exhaustion marker (PD-1) and lower expression of cytotoxicity marker (GZMB) (Fig. 7A; Table S2). It has been reported that proliferating T cells (overexpressing cyclins HMGN2, RRM2, and Ki-67) in tumors exhibit stronger exhaustion signals (PDCD1, TIGIT, and CTLA-4) (20). These findings were consistent with the notion that exhausted T cells exhibit enhanced proliferative potential and impaired cytotoxicity (20, 21). However, the immune activity of tumor-associated macrophages (Table S3), NK cells (Table S4), and CD4^+^ T cells (Table S5) was not regulated by the underlying diseases.

To verify that HBV infection indeed upregulated PD-1 expression, we included a total of 189 patients’ HCC specimens (Table S6) and detected the expression of PD-1 on CD8^+^ T cells (Fig. S12). We found that the expression of PD-1 on CD8^+^ T cells in the group with high HBsAg concentration was higher than that in the group with low one (Fig. 7B; Table S6), which was consistent with the results of HBsAg_high infiltrating CD8^+^ T cells expressing higher PD-1 than HBsAg_low (Fig. 4 to 6). Compared with HBV^negative^ HCC, CD8^+^ T cells from HBV^positive^ HCC expressed higher PD-1 (Fig. 7C; Table S6). Besides, we found that AFL, NAFL, T/N stage, and histological type could not be used as independent indicators for regulating PD-1 expression (Fig. 7C; Table S6). The results of the patients’ HCC pathological sections also verified that HBV^positive^ HCC expressed higher levels of PD-1 and TIM-3 (Fig. 7D), and HBsAg concentration was positively correlated with the expression of PD-1, but not with TIM-3 (Fig. 7D).

### Detailed leukocyte ratio was suitable for evaluating the OS

**Fig 8:**
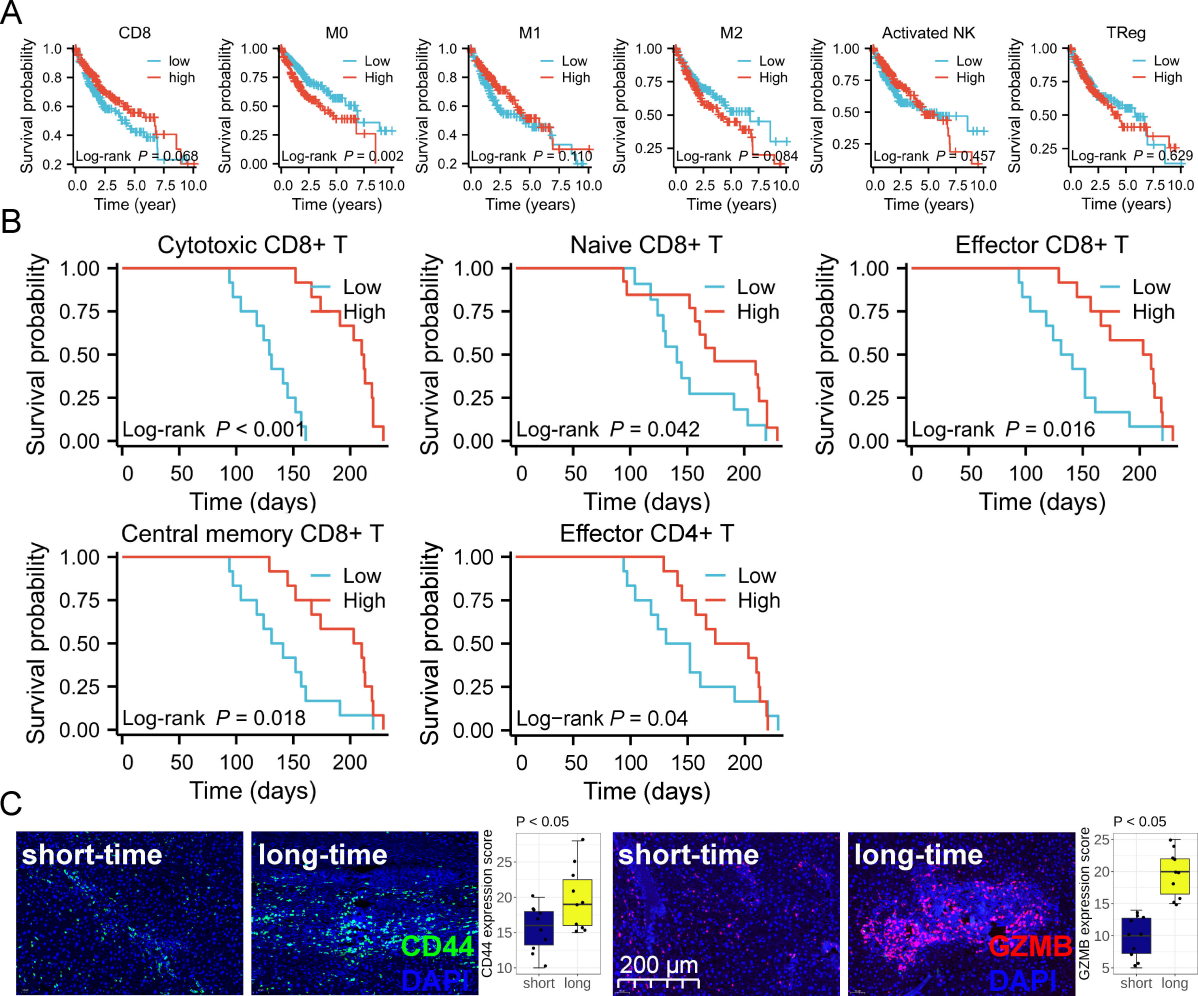
Detailed leukocyte ratio was suitable for evaluating the OS. (**A**) Kaplan-Meier curve showing the effect of CD8^+^ T cells, M0/M1/M2-like macrophages, activated NK cells, and TRegs on the OS of the patients with HCC in the TCGA database (*n =* 365). (**B**) Kaplan-Meier curve demonstrating the effect of detailed leukocyte ratio (cytotoxic CD8^+^ T cells among CD8^+^ T cells, naive CD8^+^ T cells among CD8^+^ T cells, effector CD8^+^ T cells among CD8^+^ T cells, central memory CD8^+^ T cells among CD8^+^ T cells, effector CD4^+^ T cells among CD4^+^ T cells) on the OS of the mice with HCC (*n =* 24). (**C**) Immunofluorescence showed the expression of GZMB and CD44 in the pathological sections of the mice with HCC between the long (day > 159) and short (day ≤ 159) survival time groups (*n =* 10). Statistical significance was evaluated by log-rank test (**A and B**) and Student’s *t* test (**C**).

Next, we used the Cibersort algorithm (22) to analyze the relationship between leukocyte abundance and the OS in the TCGA database (Fig. 8A). TCGA data indicated that anti-tumor leukocytes (CD8^+^ T cells, M1-like macrophages, and activated NK cells) and pro-tumor leukocytes (M2 like macrophages and TRegs) did not affect the OS of the patient with HCC (Fig. 8A). Only M0-like macrophages was detrimental to the prognosis of the patients (Fig. 8A). We believed that this inaccuracy was due to the fact that the ratio of leukocytes to non-parenchymal cells was not able to assess the prognosis of the patient. Thus, we detailed the detailed leukocyte ratio (for example, the ratio of cytotoxic CD8^+^ T cells to CD8^+^ T cells) and assessed the accuracy of the detailed leukocyte ratio prediction for the OS of the mice with HCC (Fig. 8B). It was found that: (i) the ratio of cytotoxic/naive/effector/central memory CD8^+^ T cells to CD8^+^ T cells was positively correlated with the OS (Fig. 8B); (ii) the ratio of cytotoxic CD4^+^ T cells to CD4^+^ T cells prolonged the OS (Fig. 8B). Immunofluorescence pathological slides showed that long-lived mice expressed higher GZMB and CD44 than those short-lived mice (Fig. 8C). This finding underscored the higher value of analyzing the detailed leukocyte ratio in reflecting the OS in HCC.

## DISCUSSION

In this study, we combined mass cytometry, immunofluorescence, and flow cytometry to assess the leukocyte profile in the diseased liver tissues. Our analysis revealed significant changes in T-cell, NK-cell, and macrophage function in the intrahepatic microenvironment.

First, we found that TOX, but not PD-1, was an ideal indicator of HBV-induced HCC, and PD-1 was insufficiently sensitive to this type of HCC, which explained why it did not gain sufficient benefit from anti-PD-1 antibody to some extent (5). We found that PD-1 showed limited sensitivity to HBV_HCC infiltrating T cells. The sensitivity of PD-1 to HBV_HCC was comparable to that of HCC and even worse than that of AFL_HCC/NAFL_HCC. This low sensitivity implied that the efficacy obtained with an anti-PD-1 antibody was limited. Compared with PD-1, we noticed that CTLA-4, TIM-3, and TOX showed a higher sensitivity for HBV_HCC. TOX was only induced in HBV_HCC infiltrating T cells, suggesting that combined anti-PD-1 antibody and TOX-targeting therapy better improve the anti-tumor immune activity of HBV-specific T cells. Previous studies have noticed that T cell function in peripheral blood of patients with HBV infection is not negatively correlated with serum antigen levels, and T cell function does not recover even if HBsAg is cleared (6). Our study confirmed this notion that compared with HBsAg_low, the effector/exhaustion phenotype of HBsAg_high infiltrating T cells did not change significantly, and their activity increased instead. It has been reported that CD8^+^ T cell dysfunction is not significantly correlated with PD-1 expression in HBV infection and HBV-associated HCC (7, 8). Our exploration of NK cells and T cells further refined this idea. PD-1 on NK cells was only upregulated in HBV_HCC, and the effect of HBV infection was negligible.

It is worth noting that, not only PD-1 and TOX but also other inhibitory receptors were also uniquely expressed in chronic HBV infection and HBV-associated HCC. PD-1 gradually increased with viral surface antigen titer, while TIM-3 and CTLA-4 decreased to varying degrees. When progressing to HCC, the central memory/effector/exhaustion phenotype of intrahepatic T cells was significantly enhanced, emphasizing that the immune destruction effect of HBV on T cells did not worsen with viral surface antigen titer. Similar results were found on NK cells. Based on the specific expression of these inhibitor genes, we believed that the important reason why T cell exhaustion driven by HBV was not related to PD-1 expression (7, 8) was that the induction of inhibitory receptor expression by HBV was unbalanced: PD-1 expression was upregulated, while the expression of CTLA-4/TIM-3/TOX was inhibited.

Second, we noticed that the formation of central memory T cells was inhibited in AFL and NAFL. At homeostasis, the liver contains a diverse set of non-recirculating CD8^+^ tissue-resident T cells that exhibit an activated phenotype characterized by CD69 expression and serve as local immune sentinels (23). During inflammation or an immune response to a pathogen, tissue-resident T cells acquire an interferon-secreting effector memory phenotype or a central memory phenotype important for long-term immunity (24, 25). Although CD8^+^ T cell effector and memory subsets display remarkable systemic and tissue-specific functional diversity during immune responses to pathogens, the exact role of intrahepatic CD8^+^ T cells in chronic sterile inflammatory diseases such as AFL and NAFL is still not fully understood. Current research on NAFL mainly focuses on innate immunity, and its impact on adaptive immunity is still controversial (26). We noticed that AFL and NAFL significantly antagonized the expression of inhibitory receptors on T cells and NK cells and shifted from a progenitor-like phenotype to a cytotoxic phenotype, which ensured that the cells further promoted the inflammatory response under hyperinflammation, resulting in the progressive deterioration of the disease. In addition, our study explained one of the reasons why adaptive immunity is understudied in AFL and NAFL, namely loss of central memory phenotype. AFL and NAFL cannot induce the expression of memory markers such as CD44 and CD62L on T cells and generate central memory T cells.

Finally, we believed that detailed leukocyte ratio rather than leukocyte abundance was appropriate for assessing OS in HCC. Multiple factors such as leukocyte subsets, number, spatial location, functional status, plasticity, and marker accuracy are still restricting the application of immune scoring in tumor immunotherapy and diagnosis (27). At present, there are many contradictions between immune cell infiltration and prognosis in cancers. The higher the Treg and its ratio to CD4^+^ T and CD8^+^ T cells, the shorter the OS in patients with colorectal cancer liver metastases (28). However, intra-tumoral Treg infiltration was found to be a better prognostic factor when studying advanced colon cancer patients undergoing chemotherapy or chemoimmunotherapy (29). Therefore, it is more important to determine the status of immune cells rather than the abundance. In contrast, the infiltration of immune cells in defined states is an accurate predictor of prognosis. Higher numbers of activated cytotoxic T lymphocytes are associated with improved OS and DFS (30). Infiltration of cytotoxic CD8^+^ T cells, CD3^+^ T cells, and CD45RO^+^ memory T cells in a variety of tumors is positively associated with longer DFS and improved OS (9). Our study pointed out that although the ratio of CD8^+^ T cells to non-parenchymal cells in HCC could not be a predictor for prognosis, there was a significant positive correlation between the ratio of cytotoxicity/effector/central memory CD8^+^ T cells to CD8^+^ T cells and the OS.

In conclusion, our study provides insights into in-depth knowledge of the intrahepatic microenvironment signatures across various liver diseases, which facilitates the rational design of targeted immunotherapy strategies, especially for HCC.

## MATERIALS AND METHODS

### Data sources and bioinformatics analysis

We used the Genomic Data Commons download tool (https://portal.gdc.cancer.gov) from the Cancer Genome Atlas (TCGA, *n* = 371) to download the transcriptome data and clinical information of HCC. Fragments per Kilobase million format was used to calculate transcription spectra.

### Patient specimens

From 2019 to 2023, all HCC and paired para-cancerous tissues were obtained from Fujian Medical University Union Hospital and randomly used in this experiment (*n* = 189). All patients were diagnosed with HCC based on tissue specimens. The patients had not undergone chemotherapy, radiotherapy, or other new adjuvant therapy prior to surgery. Plasma was collected from patients to detect virus-associated antigen or antibody concentrations (Table S7). According to HBsAg titer, they were divided into low, medium, and high groups with cutoff values of 25 and 250 IU/mL, respectively (Table S7).

### Construction of *in vivo* models

Wild and HBV-Tg C57BL/6J mice (male, 6 weeks old, 18−20 g) were purchased from Shanghai Model Organisms Center, Inc. and housed in a specific-pathogen-free environment with a 12/12 h day/night cycle. In order to ensure that the intrahepatic immune microenvironment is not affected by additional factors other than diseases, we employed 6-week-old male C57BL/6J mice as the model objects. Strategies for the construction of mouse liver disease models were as follows. (i) For acute liver injury, diethylnitrosamine (DEN) was supplied as a liquid vial containing 99%/10 mL, and DEN (150 mg/kg/10 mL) was diluted in sterile normal saline. Mice were injected with DEN (150 mg/kg of body weight, intraperitoneal, i.p.) for 24 h (31). (ii) For chronic liver injury, DEN was diluted in saline and injected i.p. single time at a dose of 75 mg/kg of body weight. Carbon tetrachloride (CCl_4_) was diluted 1:10 in corn oil and administered biweekly i.p. at a dose of 0.5 mL/kg of mouse for 12 weeks (32). (iii) For AFL and AFL-associated HCC, mice were fed with Lieber-DeCarli diet containing 5% (vol/vol) ethanol for 12 weeks. In order to induce HCC, the Lieber-DeCarli diet was enforced continuously for 21 months (33). (iv) For NAFL and NAFL-associated HCC models, mice were fed with a high-fat/high-cholesterol diet (HFHC, 43.7% fat, 36.6% carbohydrate, 19.7% protein, and 0.203% cholesterol) for 14 months. In order to induce HCC, HFHC was enforced continuously for 21 months (2, 34). (v) To establish liver cirrhosis, mice were injected with DEN (20 mg/kg of body weight) once a week for 2 weeks and then injected with CCl_4_ (5 mL/kg) three times per week for 6 weeks (35). (vi) The DEN and CCl_4_-induced HCC mouse model was established as previously described (36). Briefly, a single dose of DEN (25 mg/kg of body weight) was injected into mice i.p. to initiate tumor formation. Once a week with CCl_4_ (0.5 mL/kg) for an additional 16 weeks. All mice were sacrificed at 30 weeks of age. (vii) HBV-Tg mice (37) were divided into low and high HBsAg concentration groups. Chemiluminescence was performed to detect the content of virus-associated antigen or antibody in the blood (Table S8), and the titer of surface antigen was set at 150 IU/mL as a cutoff value (Table S8). (viii) For HBV-associated HCC, HBV-Tg mice were fed routinely for 12 months without additional manipulations (38). (ix) To construct natural chronic HBV infection and HBV-induced HCC models, rAAV8-1.3HBV was employed. rAAV8-1.3HBV could be used to efficiently establish a mouse model of HBV persistent infection. It is a recombinant type 8 adeno-associated virus carrying 1.3 copies of the full-length HBV genome. It combines the characteristics that the HBV genome with repetitive regions can replicate in the mouse liver with the characteristics of AAV8 hepatophilic cells and is employed to establish the HBV chronic infection model. Moreover, the expressions of HBV DNA, HBeAg, and HBsAg are continuously detected in mouse liver and blood (39 – 41). rAAV8-1.3HBV plasmid was diluted to 8 µg/mL with saline, and HBV plasmid (0.5, 1, 2, and 3 mL) was injected into the mice through the tail vein. Blood was collected from the tail vein after 6 weeks to detect virus-associated antigen or antibody concentration (Table S9). According to the HBsAg titer, they were divided into low, medium, and high groups with cutoff values of 100 and 500 IU/mL, respectively (Table S9). Mice constructed with rAAV8-1.3HBV (8 µg/mL, 3 mL) were reared normally for 10–11 months, and subsequent experiments were carried out after they developed into HCC. (x) The control group did not receive any treatment. (xi) The growth status, mental state, appetite, and body weight of the mice in the control and treatment groups were observed. All models were identified by HE staining to judge whether the modeling was successful.

### Measurement of HBV markers

Chemiluminescent microparticle immunoassay was used to detect the anti-HBsAg, HBeAg, anti-HBeAg, HBcAg, and anti-HBcAg in patient and mouse serum using the Architect i2000 automated chemiluminescence immunoassay instrument, according to the manufacturer’s instructions. If the level of HBsAg in the sample was >250 IU/mL, the sample was diluted with normal saline to ensure that the HBsAg was <250 IU/mL.

### ELISA detection of liver function-related indicators

The standard of ALT, AST, ALP, and GGT was diluted. Forty microliters of the samples or standards was added to the wells and incubated at 37°C for 30 min. They were washed three times with PBS buffer, followed by the addition of 50 µL of enzyme-labeled antibody, and incubated at RT for 30 min, and then 50 µL of chromogenic solution and 50 µL of stop solution were added. The absorbance value of each sample at a wavelength of 450 nm was measured with Multiskan FC System.

### Harvesting and processing of mouse liver samples

Mice were euthanized with carbon dioxide (CO_2_) and perfused with PBS buffer through the left ventricle of the heart using a 25-G butterfly needle attached to a 50 mL syringe (42). The collected complete liver sample was dissected into approximately 1–2 mm-diameter pieces using scissors and digested (0.4 mg/mL collagenase from *Clostridium histolyticum*, type IV [collagenase IV], 10 mg/mL deoxyribonuclease I from bovine pancreas [DNase I], 10% fetal bovine serum [FBS], and Roswell Park Memorial Institute [RPMI] 1640 medium) at 37°C for 30 min, applying continuous shaking. The enzymatic reaction was stopped by adding ethylenediaminetetraacetic acid (EDTA) in PBS buffer to a final concentration of 5 mM. To homogenize the sample, it was repeatedly aspirated and ejected using a 5 mL syringe with a 20-G needle until a uniform homogenate was formed (42). Afterward, the homogenate was filtered through a 70 µm cell strainer and centrifuged at 400 × g for 8 min at 4°C to pellet the cells and myelin. This was followed by the myelin removal step by gradient centrifugation with 30% Percoll in PBS buffer (1,592 × g for 30 min at 4°C; without brakes during deceleration) using a 50 mL tube with a lid for a fixed angle rotor fitting in a centrifuge. After myelin (the top white layer) separation, the middle transparent layer without the bottom layer of red blood cells was collected and filtered through a 70 µm cell strainer. The single-cell suspension was washed in PBS buffer and centrifuged at 400 × g for 8 min at 4°C to pellet the cells. Cells were then ready for cytometry analysis.

### Processing of patient samples for cytometry analysis

Fresh resected liver tissue samples were taken within 2 h to the laboratory to start tissue dissection and processing. First, the tissue samples were thoroughly washed with PBS buffer to remove visible blood clots and to reduce blood leukocyte contamination. Then, the tissues were minced using scalpels into approximately 3–5 mm-diameter pieces and digested (1 mg/mL collagenase IV, 10 mg/mL DNase I, 10% FBS, and RPMI 1640) at 37°C for 45 min using the gentle MACS Octo Dissociator with Heaters and continuous shaking. The enzymatic reaction was stopped by adding 2 mM EDTA in PBS buffer to double volume of the sample. Afterward, the homogenate was filtered through a 100 µm cell strainer and centrifuged at 400 × *g* for 8 min at 4°C to pellet the cells and myelin. This was followed by the myelin removal step by gradient centrifugation with 30% Percoll in PBS (1,592 × *g* for 30 min at 4°C; without brakes during deceleration) using a 50 mL tube with a lid for a fixed angle rotor fitting in a centrifuge. After myelin (the top white layer) separation, the middle transparent layer without the bottom layer of red blood cells was collected and filtered once more through a 100 µm cell strainer. The single-cell suspension was washed in PBS buffer and centrifuged at 400 × *g* for 8 min at 4°C to pellet the cells. Next, the cells were ready for cytometry analysis.

### Metal-isotope-tagged antibodies

Pre-conjugated antibodies to metal isotope were purchased from Fluidigm in purified form and conjugated in-house using the Maxpar X8 chelating polymer kit according to the manufacturer’s instructions.

### Cell surface staining for cytometry

To avoid nonspecific binding of antibodies, the sample was incubated at 4°C for 15 min in Human TruStain FcX (Fc Receptor Blocking Solution) or TruStain FcX (anti-mouse CD16/32) Antibody. Without washing, the cells were spun down, resuspended in the antibody mixture in PBS buffer, and incubated at 4°C for 30 min. After staining the surface antibodies for mass cytometry, we added Cell-ID Cisplatin to the sample for 3 min to discriminate viable/dead cells. Then, the sample was washed once in PBS buffer and centrifuged to pellet the cells. In order to identify HBV-specific T cells, cells were simultaneously stained for HBsAg-specific responses, as described. The negative condition was used to subtract any background staining.

### Intracellular cytokine staining for cytometry

Cells were permeabilized using FOXP3 Fix/Perm Buffer Set according to the manufacturer’s instructions for 45 min at 4°C. Subsequently, the sample was washed once in Perm/Wash buffer and incubated in the antibody mixture in Perm/Wash buffer for 30 min at 4°C. The sample was washed once in Perm/Wash buffer and centrifuged to pellet the cells.

### Cell preparation and cytometry acquisition

For mass cytometry, after cell surface and intracellular antibody staining, the cells were incubated in 4% paraformaldehyde aqueous solution overnight. Prior to the acquisition, the cells were pelleted without washing and resuspended in up to 1 mL of diluted 1:3,000 Cell-ID Intercalator-Ir + Maxpar Fix and Perm buffer for 3 h. After the sample was washed twice in PBS buffer and twice in ddH_2_O, the sample was diluted to 1.5 × 10^6^ cells/mL in ddH_2_O containing 10% EQ Four Element Calibration Beads and filtered through a 40 µm filter cap FACS tube. Samples were analyzed with a Helios Cytometry by time-of-flight (CyTOF)2. Quality control and tuning processes on the Helios CyTOF2 were performed following the guidelines for the daily instrument operation. For flow cytometry, samples were analyzed using FACSCelesta Flow Cytometer.

### Removal of dead and dying cells

Leukocytes were collected and centrifuged at 300 × *g* for 5 min. Supernatants were removed and the cells were resuspended in 100 µL of dead cell-removal beads per 1 × 10^7^ cells as described by the manufacturer. The mixture was incubated at RT for 15 min and added to the LS column. The columns were then washed four times with binding buffer. Live cells were collected from the flow-through.

### Tissue collection for immunofluorescence

A portion of the tissues was collected for immunofluorescence. The samples were cut into pieces of max 1 cm × 0.5 cm × 0.5 cm and embedded in cryomolds filled with cryo embedding medium. Freezing was performed in a beaker filled with 2-methylbutane on dry ice directly and stored at −80°C until further processing.

### Immunofluorescence

Sections were fixed with 4% paraformaldehyde aqueous solution, washed in PBS buffer, and incubated with a blocking solution consisting of PBS buffer supplemented with 1% bovine serum albumin and 0.3% Triton X-100. Subsequently, sections were incubated with the following primary antibodies (diluted in blocking solution) at 4°C overnight. Sections were then washed with blocking solution and incubated with secondary antibodies and one of the following directly labeled antibodies (all diluted in blocking solution) at RT for 2 h. Sections were washed with blocking solution and incubated in Sudan black B dissolved in 70% ethanol to reduce autofluorescence of the tissues at RT for 30 min. Finally, sections were washed with Hanks Balanced Salt Solution buffer and mounted with Antifading Mounting Medium with 4′,6-diamidino-2-phenylindole. Fluorescence photomicrographs were captured with an Olympus IX81 microscope (43).

### Preprocessing of cytometry data

Raw mass cytometry data were normalized using the MATLAB version of the Normalizer tool (44). Cells were assigned by manually gating on Event length and DNA (^191^Ir and ^193^Ir) channels, followed by the dead cell discrimination analyzing ^195^Pt expression using FlowJo Software. Doublets were excluded using Gaussian discrimination channels. Next, data were concatenated and de-barcoded using Boolean gating in FlowJo software. The normalized data containing living cells from every individual sample were manually exported from FlowJo Software and imported into R studio of R using the R packages “flowCore” (45) and “flowWorkspaceData” (46) (R Foundation for Statistical Computing). Before automated high-dimensional data analysis, the mass cytometry data were transformed with a cofactor in the range of 5 and 60 using an inverse hyperbolic sine function (47). Then, live, single, CD3-positive, and compensated cells were exported and imported into R Studio. Additionally, all cytometry data were normalized between 0 and 1 to the 99–999th percentile of the merged sample in each batch.

### Automated subset identification

To identify T cell subsets accurately, we first carried out a step of Flow or mass cytometry data using a Self-Organizing Map (FlowSOM) clustering (48) to generate a starting point of 100 nodes, on pre-processed and combined mass cytometry data sets (15, 49). This was then followed by expert-guided manual meta-clustering using parameters. The respective *k*-value was manually chosen (in the range of between 20 and 30); identified subsets were annotated and merged based on a similarity of marker expression in order to uphold the biological relevance of the data set. Manually annotated subsets were used to calculate the relative frequencies of immune cell subsets. Heatmaps display median expression levels of all markers per merged subset and are plotted using the R package “pheatmap.” From mass cytometry data sets, we pre-selected major subsets and performed additional FlowSOM analysis to identify smaller cell subsets. We calculated the median marker expression among selected cell types of the mass cytometry batch using the R package “dplyr.” For data visualization, we applied dimensionality reduction techniques. For a complex overview of the immune compartment, we used t-SNE (50). To create a t-SNE of isolated T cells, we pooled equally proportioned 12,000 T immune cells from the data sets from the CyTOF batch. Categorical One-SENSE analysis generated one-dimensional t-SNE of equally pooled T cells of the CyTOF batch, where the axis was calculated using inhibition or activation markers. The one-dimensional t-SNEs were aligned with two heatmaps, displaying inhibition or activation cell profile using the R package “gplots.”

### Statistical analysis

*P*-values were calculated to compare the relative frequencies of leukocyte subsets or median marker expression among different groups using one-way ANOVA or the Kruskal-Wallis test. All data were presented as mean ± standard deviation (χ ± s), reported *P*-values were below 0.05, considered statistically significant, and displayed on the corresponding graph. Relative frequencies of leukocytes or median marker expression were generated using the R package ggplot2. When comparing two samples, the independent sample *t* test was used if the data were normally distributed, and the variances were homogeneous. If the data were normally distributed with unequal variance, the Wilcoxon rank sum test was used. Log-rank test was used to compare the difference between the two survival curves. Pearson’s correlation matrix between the relative frequencies of immune populations was calculated with the R environment (“Hmisc” R package) and includes *P*-value and correlation coefficients. Correlations were considered statistically significant if the *P*-value was below 0.05 and *R* value was below −0.9 or above 0.9 and visualized using the R package “circlize” (51).

## SUPPLEMENTAL MATERIAL

Supplemental Material spectrum.02860-23-s0001.pdf Supplemental figures, tables, and materials.

ASM does not own the copyrights to Supplemental Material that may be linked to, or accessed through, an article. The authors have granted ASM a non-exclusive, world-wide license to publish the Supplemental Material files. Please contact the corresponding author directly for reuse.
