# Supplementary material for: Single-cell atlas reveals characteristic changes in intrahepatic HBV-specific leukocytes
Source: Microbiol Spectr. 2023 Nov 30;12(1):e02860-23. doi: 10.1128/spectrum.02860-23 (PMC10782979; doi:10.1128/spectrum.02860-23)
Supplement: Supplemental Material — Supplemental figures, tables, and materials. [file spectrum.02860-23-s0001.pdf]

Supplemental Information for

Single-cell atlas reveals characteristic changes in intrahepatic HBV-specific leukocytes

Banglun Pan, Zengbin Wang, Rui Chen, Xiaoxia Zhang, Jiacheng Qiu, Xiaoxuan Wu, Yuxin Yao, Yue Luo, Xiaoqian Wang, Nanhong Tang

Table of contents

SUPPLEMENTARY FIGURES 2

SUPPLEMENTARY TABLES 13

SUPPLEMENTARY MATERIALS 32

SUPPLEMENTARY REFERENCES 45

SUPPLEMENTAL FIGURES

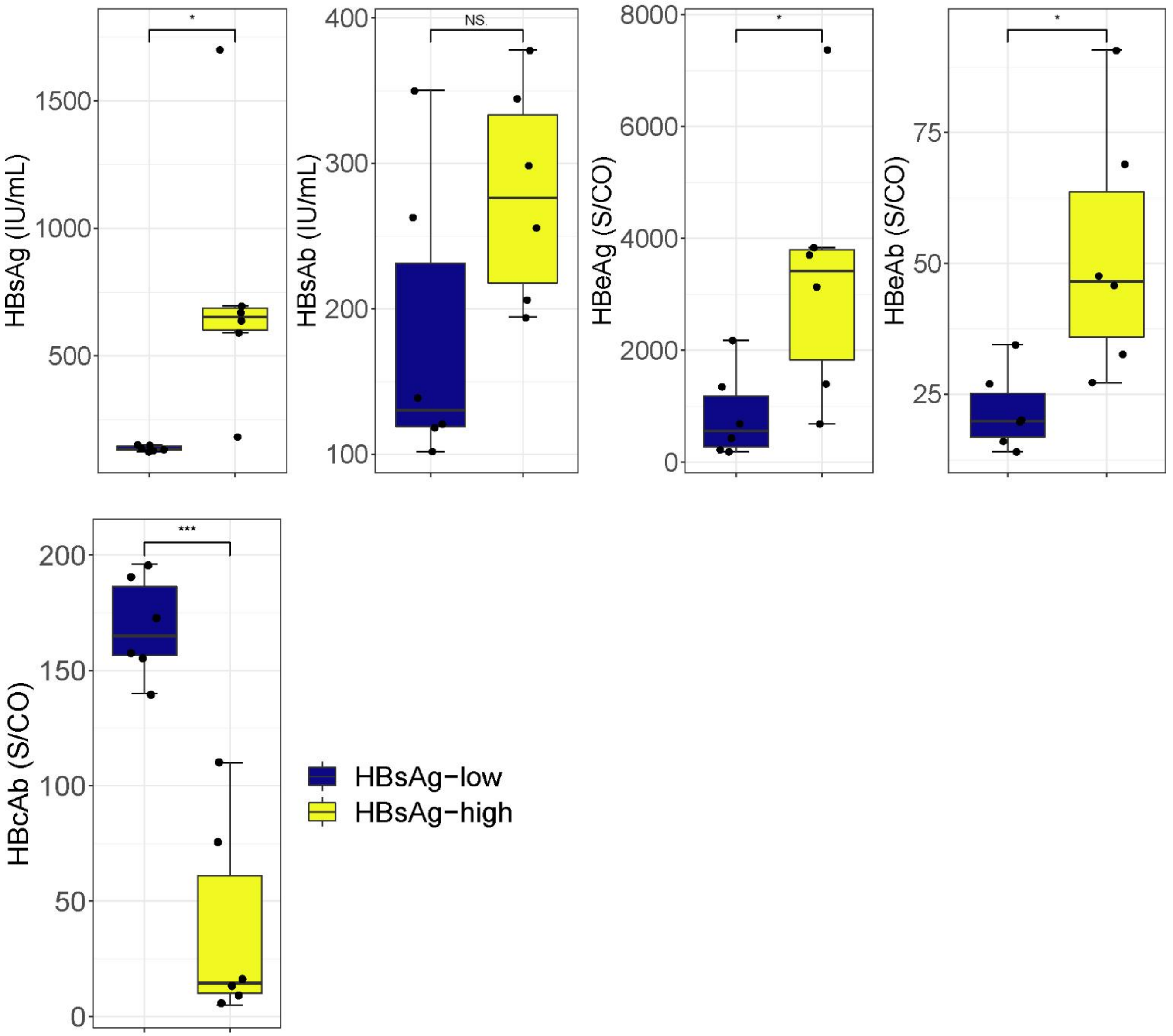

Figure S1. The content of HBsAg, HBsAb, HBeAg, HBeAb, and HBcAb in blood of HBV-Tg mice from the *HBsAg_low* and *HBsAg_high* groups with cut-off value of 150 IU/mL (*n* = 6). Mean ± SD. Statistical significance evaluated by Student's *t* test. **P* < 0.05, ***P* < 0.01, ****P* < 0.001.

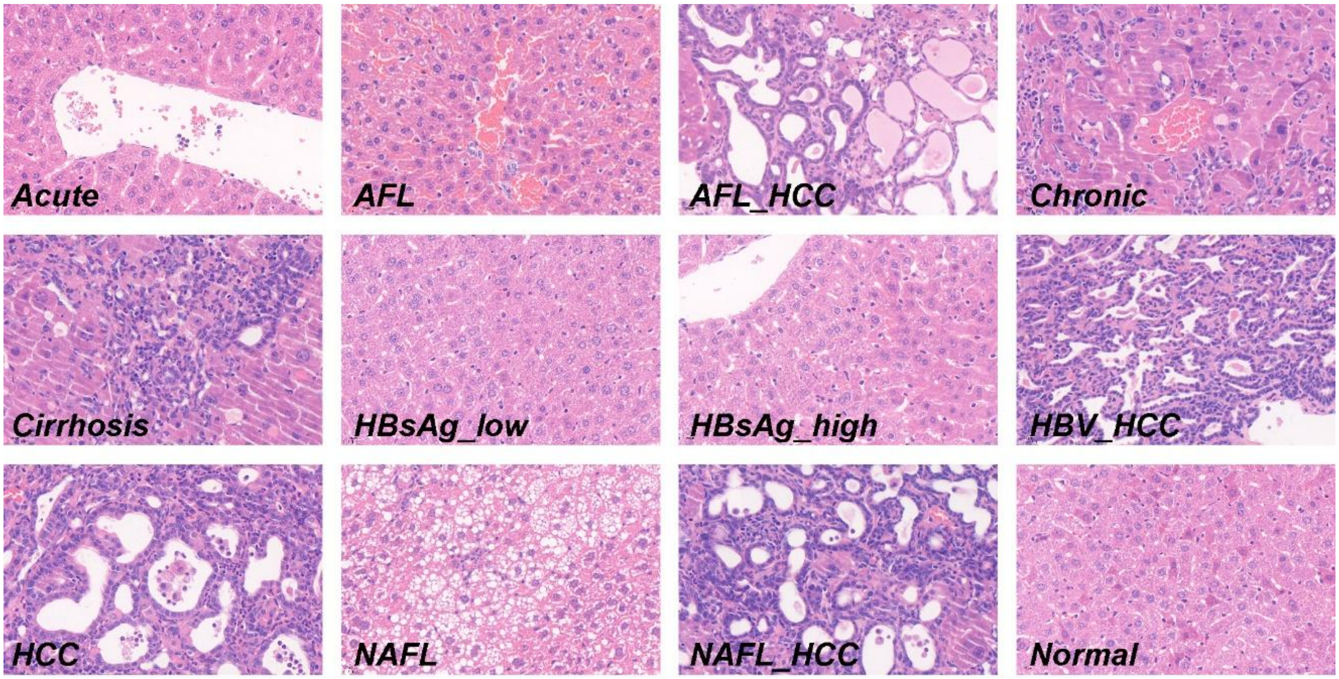

Figure S2. HE staining identified 12 mouse models. Including *Acute*, *AFL*, *AFL_HCC*, *Chronic*, *Cirrhosis*, *HBsAg_low*, *HBsAg_high*, *HBV_HCC*, *HCC*, *NAFL*, *NAFL_HCC*, and *Normal* tissues (*n* = 6).

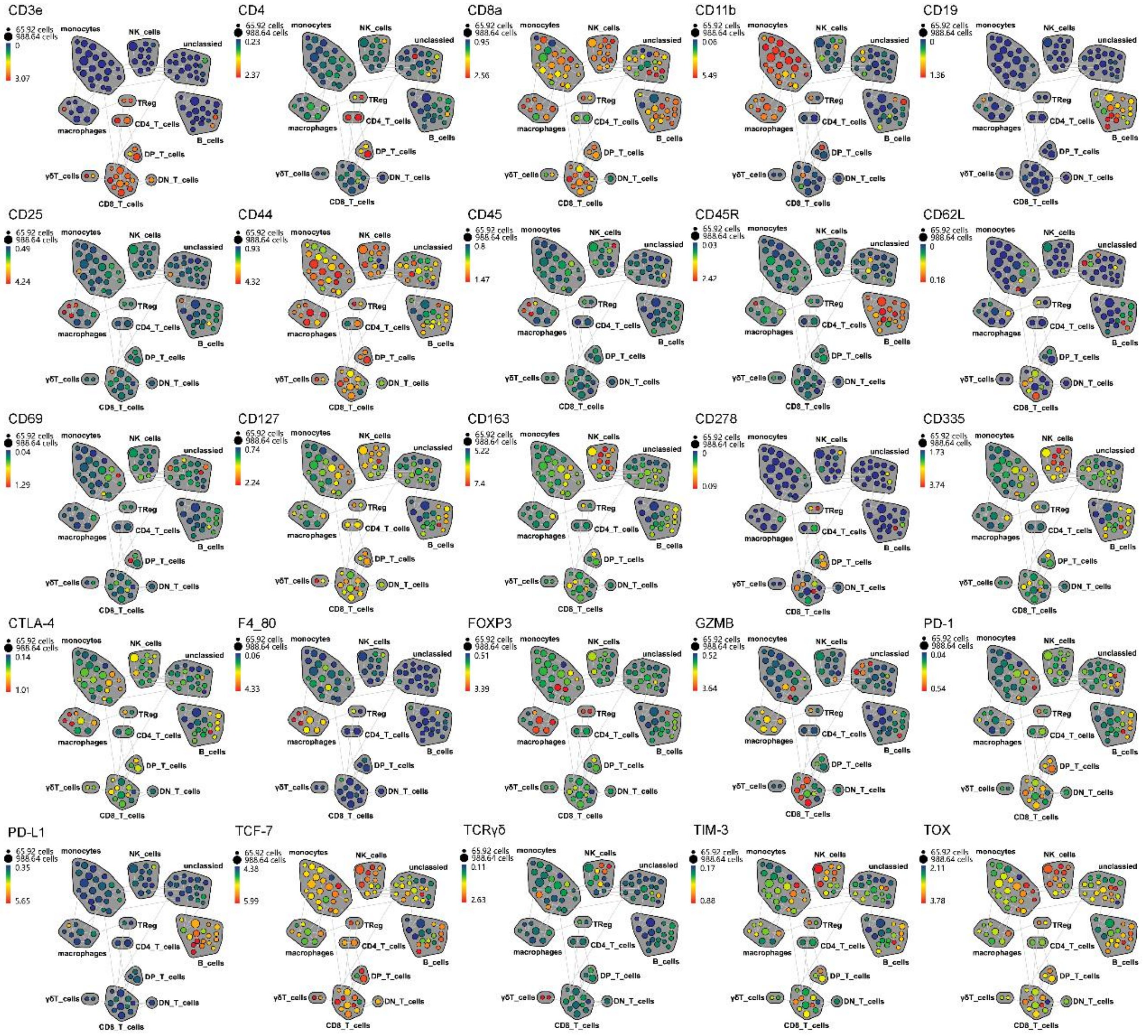

Figure S3. SPADE cluster plot calculated by mouse intrahepatic CD45$^+$ leukocytes overlaid with all markers (n = 72).

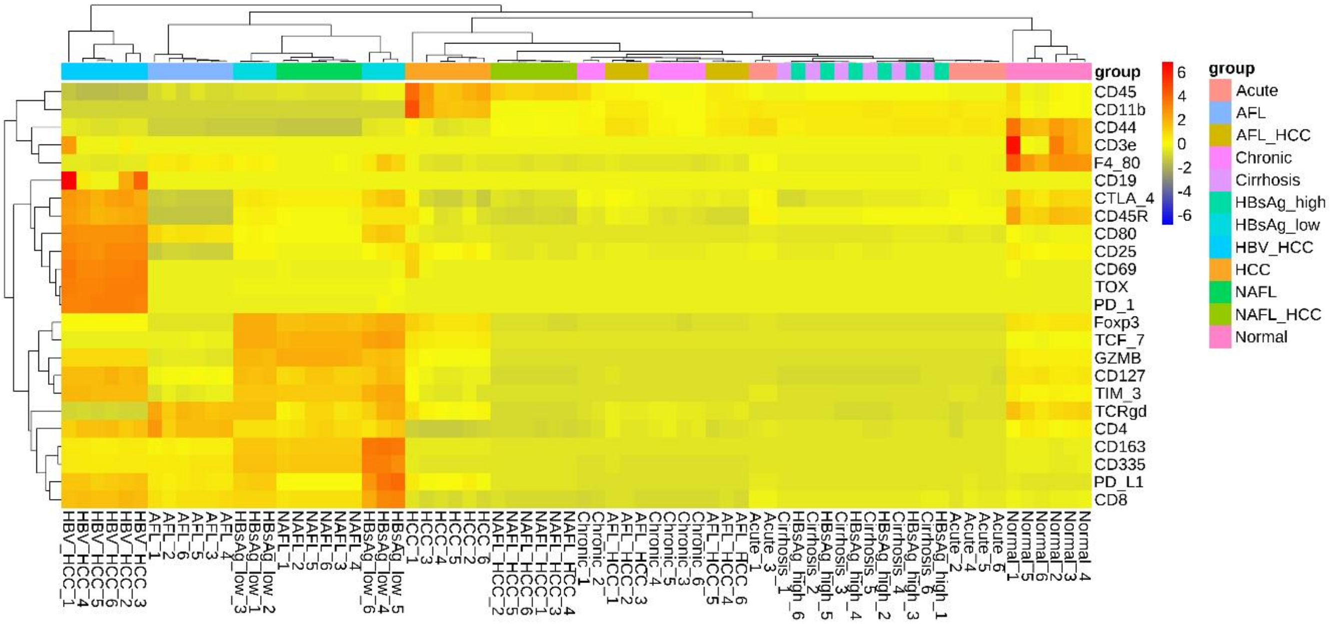

Figure S4. Heatmap showing mean expression of all markers calculated by mouse intrahepatic CD45⁺ leukocytes (*n* = 72).

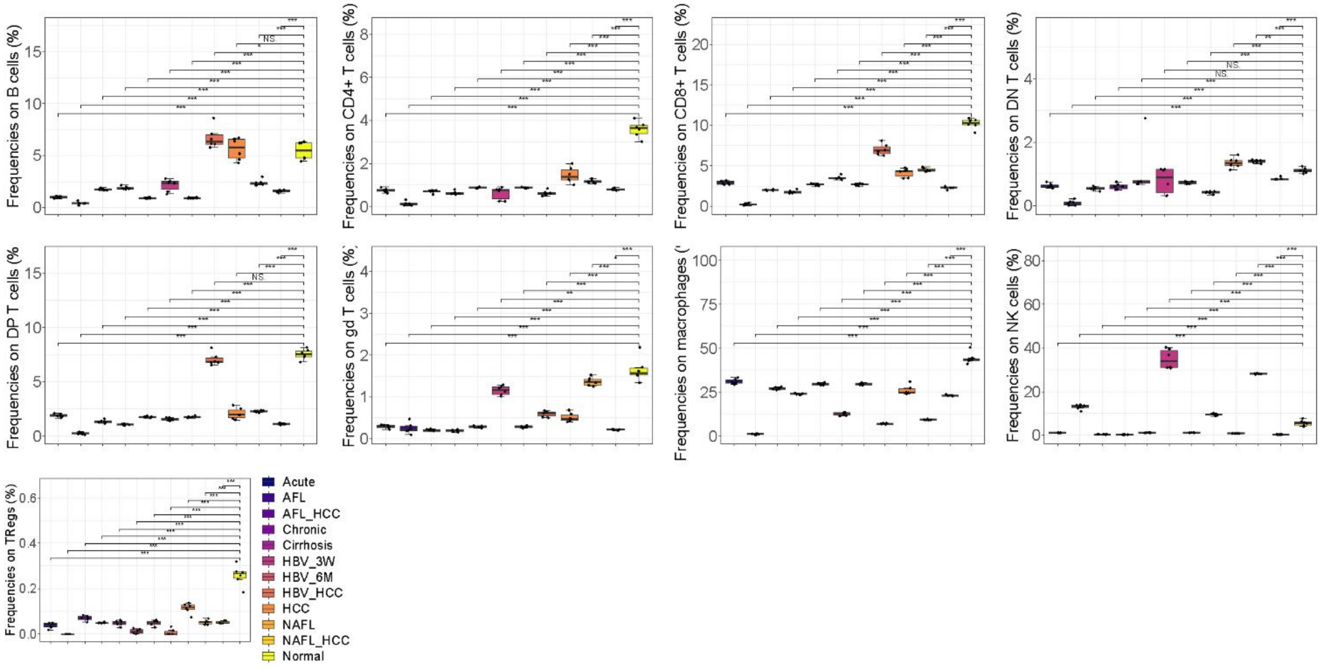

Figure S5. Mass cytometry analysis of unique changes in the intrahepatic leukocytes

across different liver models. Relative frequency of mouse intrahepatic leukocyte subsets in diseased and normal livers (*n* = 6). Mean ± SD. Statistical significance evaluated by Student's *t* test. **P* < 0.05, ***P* < 0.01, ****P* < 0.001.

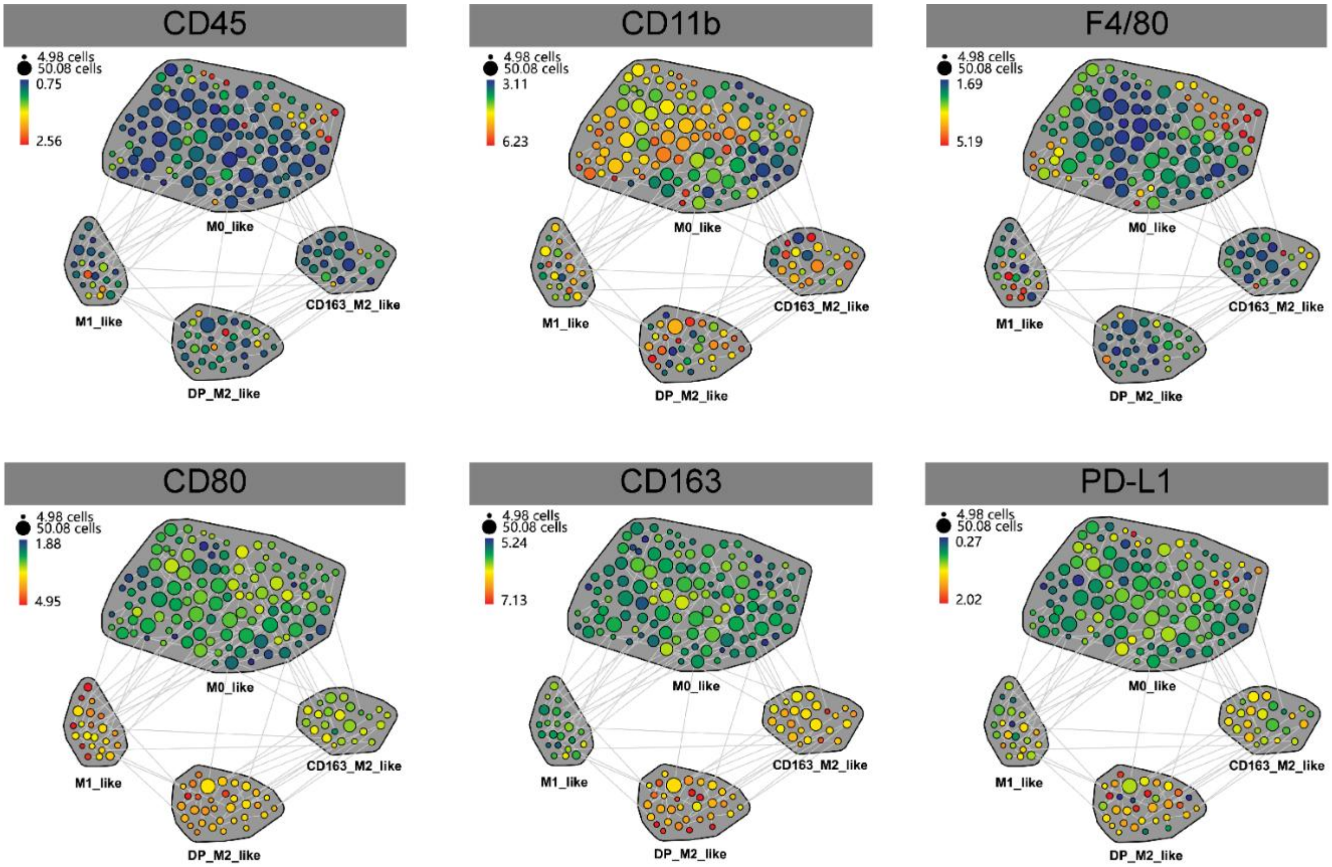

Figure S6. A single SPADE cluster plot calculated by mouse intrahepatic macrophages overlapped with CD45, CD11b, F4/80, CD80, CD163, PD-L1 (*n* = 72).

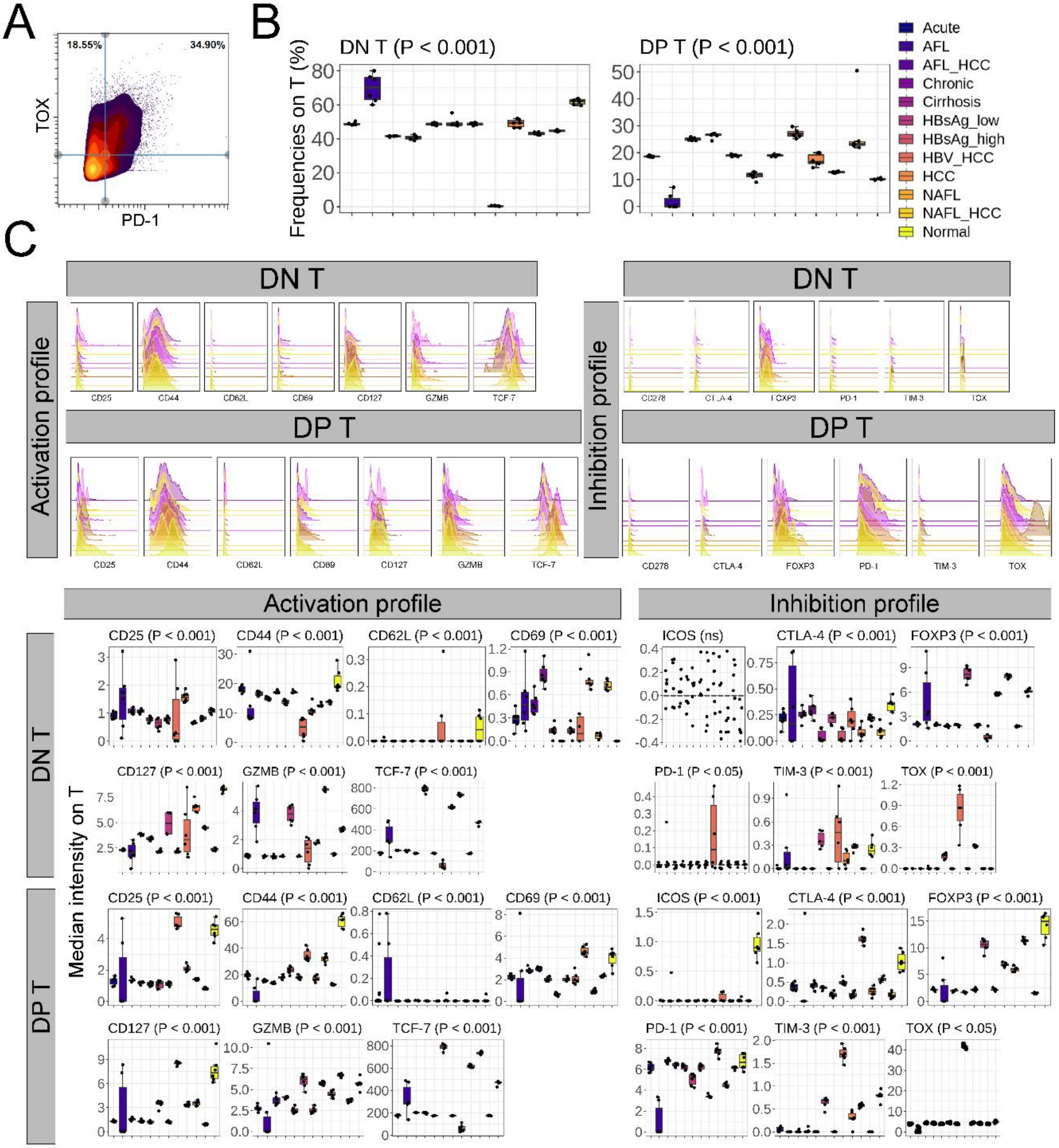

Figure S7. Characterization of the exhaustion and effector phenotypes of mouse intrahepatic T cells. (A) Gating strategy for mouse intrahepatic PD-1^{positive} TOX^{positive} (double positive, DP) and PD-1^{negative} TOX^{negative} (double negative, DN) T cells. (B) Relative abundance

of mouse intrahepatic T cells subsets in different liver diseases (n = 6). (C) Representative peak plots and boxplots showing differentially expressed markers on mouse intrahepatic DN or DP T (n = 6). Mean ± SD. Statistical significance determined by one-way ANOVA or Kruskal-Wallis test (B-C).

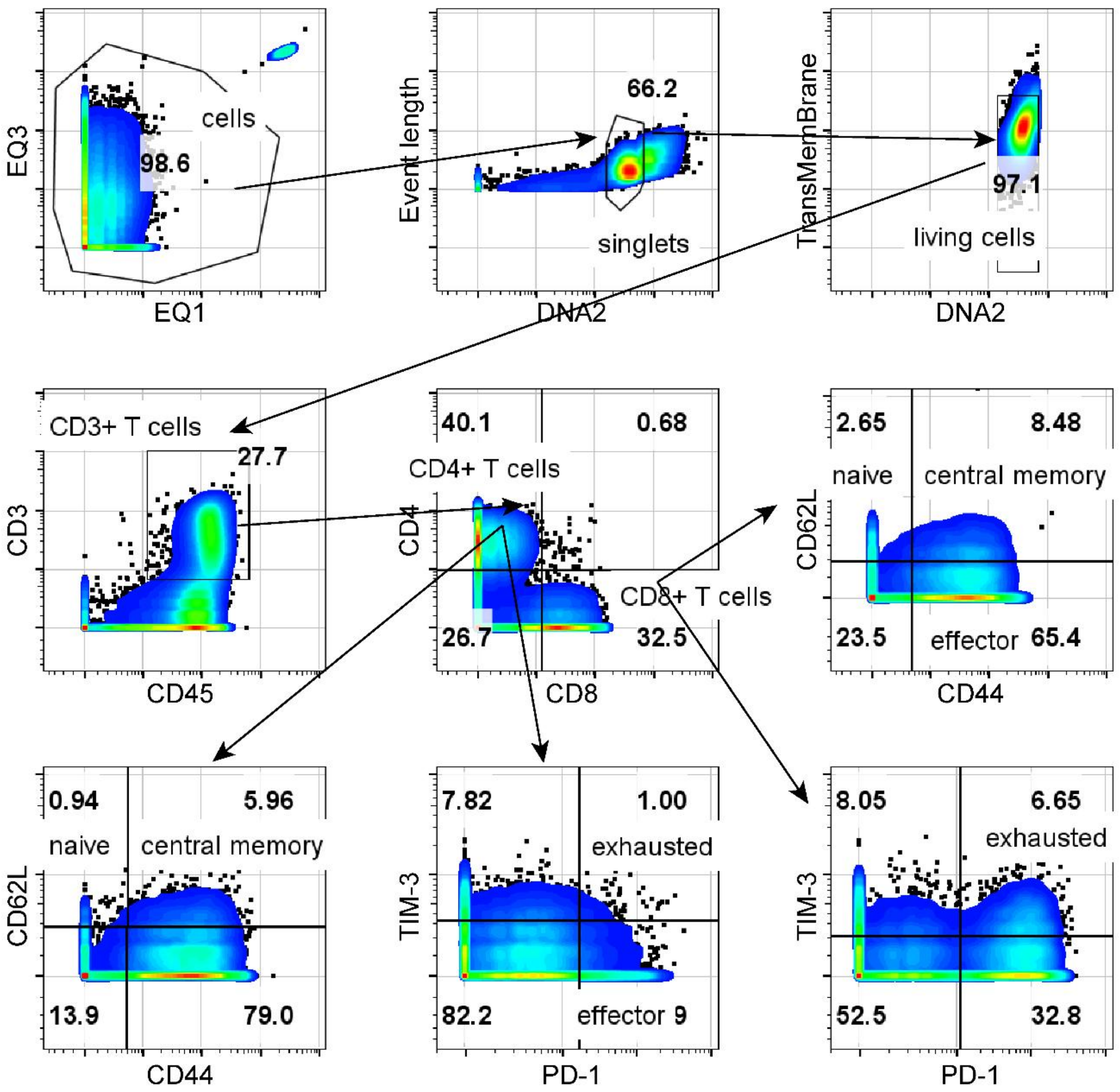

Figure S8. Manual gating strategy to identify mouse intrahepatic naïve/effector/central memory/exhausted CD4⁺/CD8⁺ T cell subsets.

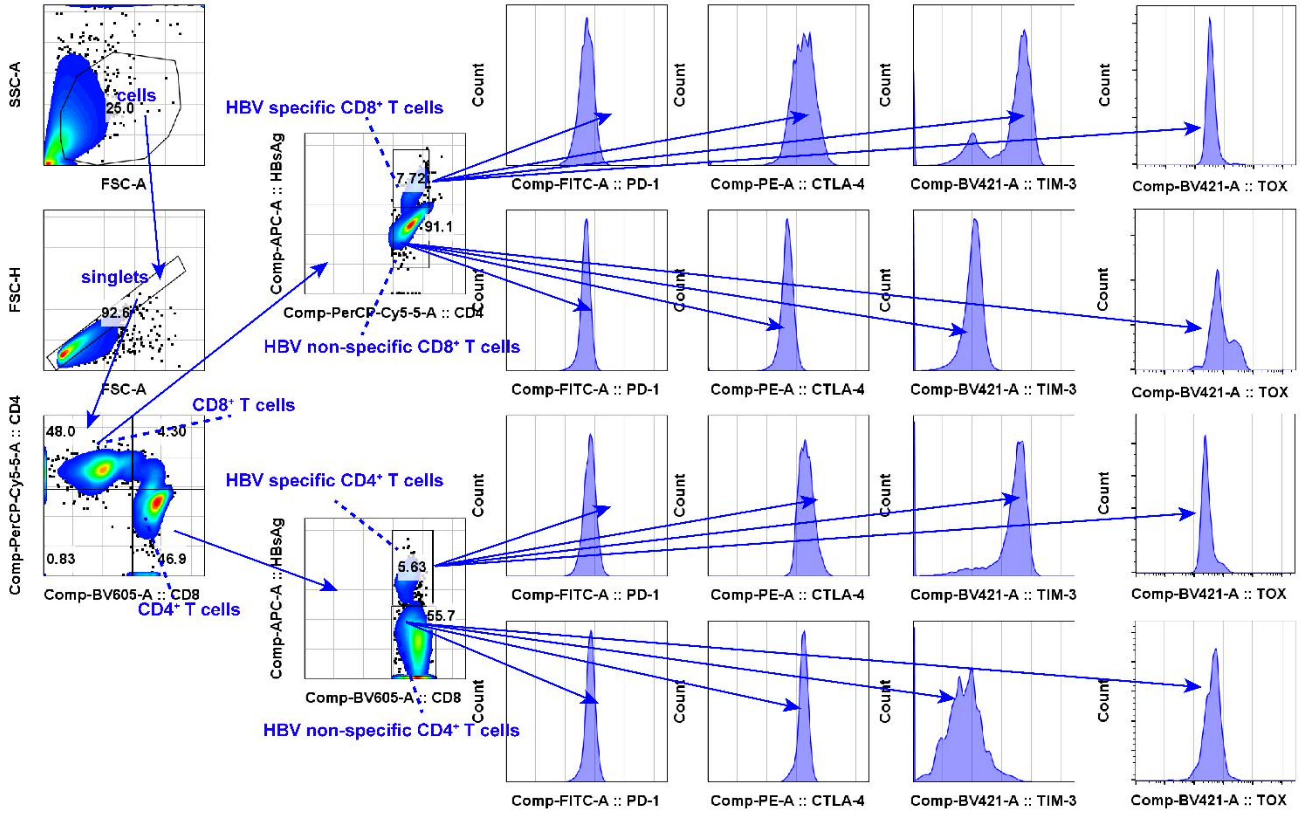

Figure S9. Manual gating strategy to identify PD-1, CTLA-4, TIM-3, and TOX expression on mouse or patient intrahepatic HBV specific and HBV un-specific CD4⁺ and CD8⁺ T cells.

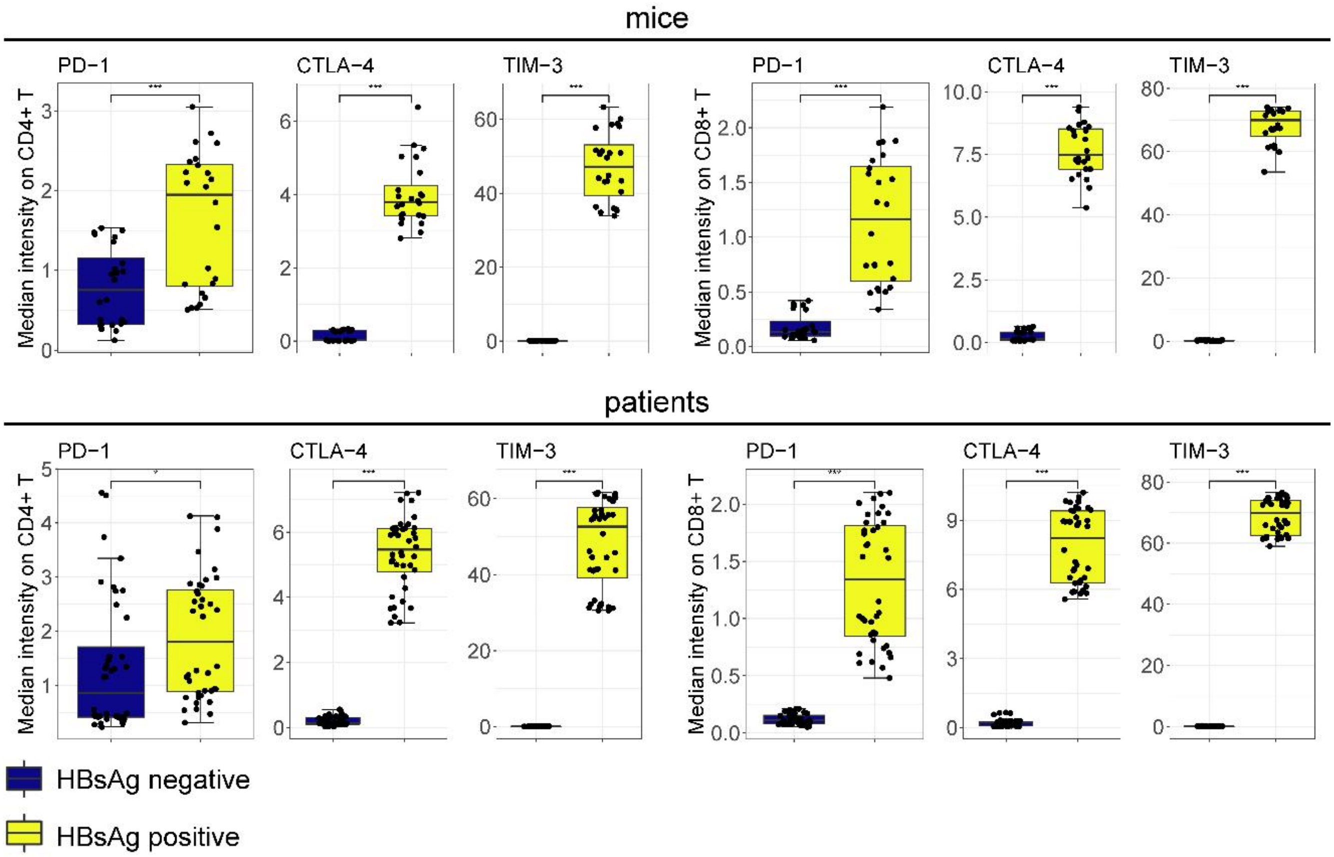

Figure S10. Expression of PD-1, CTLA-4, and TIM-3 on HBV-specific and HBV non-specific $CD4^+$ and $CD8^+$ T cells in mice (*n* = 6) and patients (*n* = 10). Mean ± SD. Statistical significance evaluated by Student's *t* test. *$P < 0.05$, **$P < 0.01$, ***$P < 0.001$.

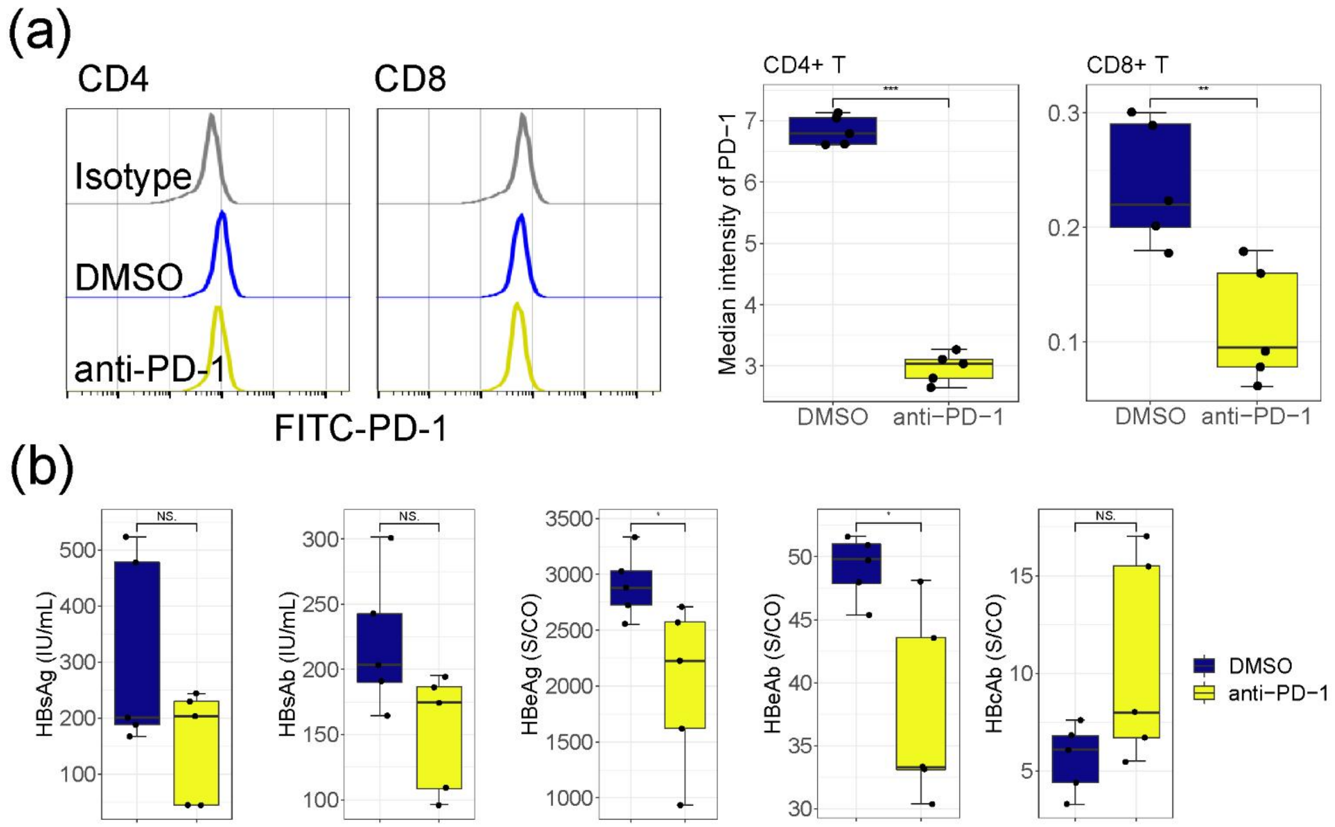

Figure S11. Effect of PD-1 inhibition on HBV-associated HCC infiltrating T cells. (A) Inhibitory receptor expression on $CD4^+$ and $CD8^+$ T cells from the DMSO and anti-PD-1 antibody groups (n = 5). (B) The content of HBsAg, HBsAb, HBeAg, HBeAb and HBcAb in blood from the DMSO and anti-PD-1 antibody groups (n = 5). Mean ± SD. Statistical significance evaluated by Student's t test (A-B). *$P < 0.05$, **$P < 0.01$, ***$P < 0.001$.

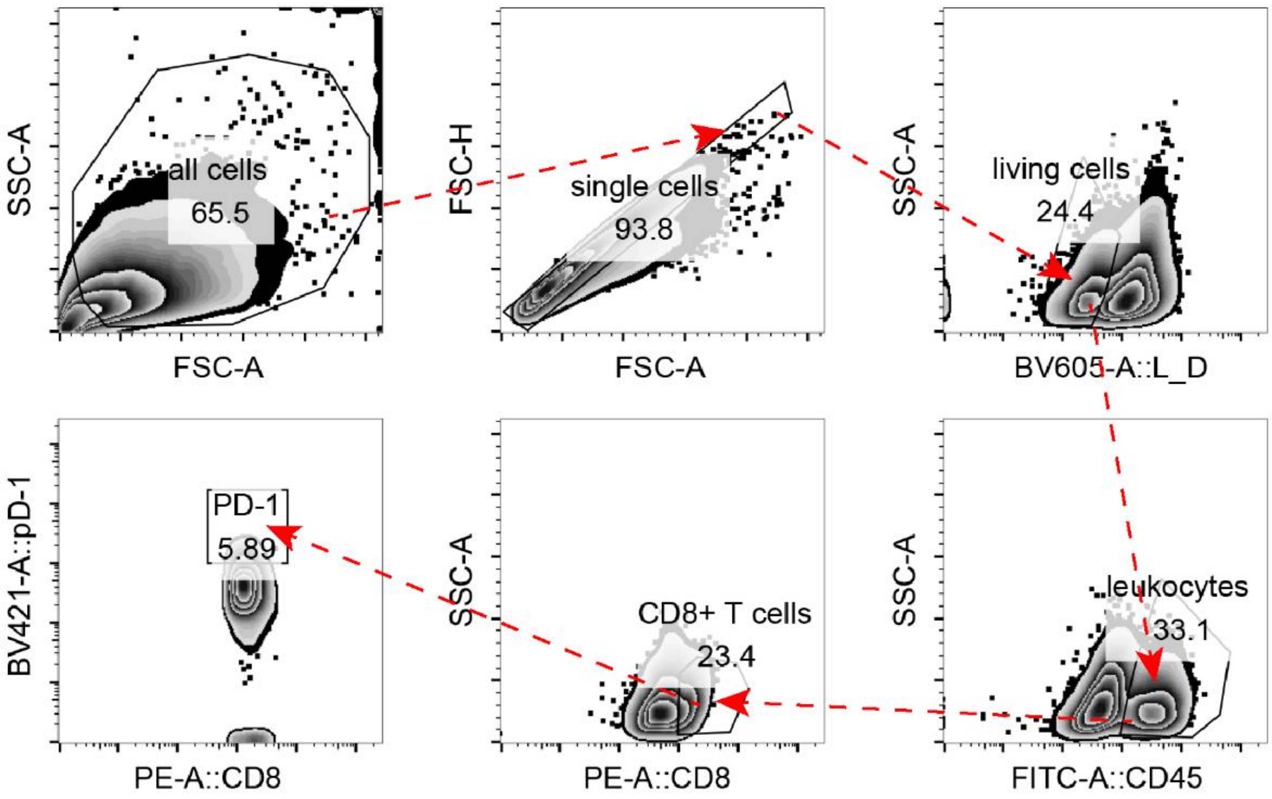

Figure S12. Gating strategy to analyze PD-1 expression on patient HCC infiltrating CD8⁺ T cells.

SUPPLEMENTAL TABLES

Table S1. Relative abundance of patient HCC infiltrating CD45⁺ leukocyte subsets

Frequencies(%) / Groups	B	NK	NKT	CD4⁺ T	CD8⁺ T	DN T	DP T	mono cyte	macro phage
No background HCC	6.99	1.11	0.36	22.91	19.61	2.46	1.01	5.84	6.49

AFL_HCC	1.15	1.07	0.21	30.98	16.19	1.56	1.73	18.90	23.04
NAFL_HCC	13.09	3.22	1.08	11.50	7.12	1.94	0.37	5.53	11.20
HBV_HCC	3.17	0.89	0.51	15.04	9.96	0.58	9.25	29.79	32.40
HBV_AFL_HCC	4.03	1.36	0.17	32.94	20.02	2.62	1.70	10.40	15.35
HBV_NAFL_HCC_1	5.54	3.18	0.30	31.68	20.10	4.51	1.56	9.16	8.96
HBV_NAFL_HCC_2	2.51	0.57	0.15	20.92	16.01	4.04	2.71	14.18	15.93

Abbreviation: HCC, hepatocellular carcinoma; AFL, alcoholic fatty liver; NAFL, non-alcoholic fatty liver; HBV, hepatitis B virus; NK, natural killer; DN, double negative; DP, double positive.

Table S2. Expression of restricted markers on patient HCC infiltrating $CD8^+$ T cells

MFI / Groups	CD103	CD127	CD25	CD278	CD38	CD80	CD86	CTLA-4	GZMB	Ki-67	LAG-3	OX40	PD-1	TIGIT	TIM-3
No background HCC	0.00	0.85	0.00	2.41	5.24	0.00	0.00	0.00	40.77	0.41	0.20	0.00	0.00	2.99	0.00
AFL_HCC	0.01	0.03	0.00	1.70	4.44	0.00	0.00	0.00	45.20	0.59	0.23	0.00	0.00	5.04	0.00
NAFL_HCC	0.00	0.00	0.00	1.49	9.49	0.00	0.00	0.00	50.38	0.36	0.00	0.00	0.00	5.98	0.00

HBV_HCC	1.07	8.16	0.00	1.51	21.80	0.00	0.00	0.00	35.63	1.59	0.73	0.00	0.18	2.25	0.00
HBV_AFL_HCC	0.00	3.39	0.00	2.28	14.86	0.00	0.00	0.00	33.55	0.87	0.06	0.00	0.50	2.88	0.00
HBV_NAFL_HCC_1	0.00	39.32	0.00	1.92	2.44	0.00	0.00	0.00	21.29	1.28	0.18	0.00	0.66	0.56	0.00
HBV_NAFL_HCC_2	0.73	15.74	0.00	1.30	3.40	0.00	0.00	0.00	23.28	1.11	0.13	0.00	0.90	5.85	0.00

Abbreviation: HCC, hepatocellular carcinoma; AFL, alcoholic fatty liver; NAFL, non-alcoholic fatty liver; HBV, hepatitis B virus; MFI, mean fluorescence intensity; CTLA-4, cytotoxic T lymphocyte-associated antigen-4; GZMB, granzyme B; LAG-3, lymphocyte-activation gene 3; PD-1, programmed cell death protein 1; TIGIT, T cell immunoreceptor with Ig and ITIM domains; TIM-3, T-cell immunoglobulin and mucin-domain containing-3.

Table S3. Expression of restricted markers on patient HCC infiltrating macrophages

MFI / Groups	CD80	CD86	CD206	CD163	PD_L1
No background HCC	0.00	6.33	0.15	1.51	0.00
AFL_HCC	0.00	7.59	0.28	4.17	0.10
NAFL_HCC	0.00	4.75	0.17	0.22	0.05
HBV_HCC	0.00	3.44	0.03	0.64	0.10
HBV_AFL_HCC	0.00	6.47	0.59	1.68	0.00

HBV_NAFL_HCC_1	0.00	7.91	0.54	3.24	0.12
HBV_NAFL_HCC_2	0.00	2.61	0.00	0.39	0.00

Abbreviation: HCC, hepatocellular carcinoma; AFL, alcoholic fatty liver; NAFL, non-alcoholic fatty liver; HBV, hepatitis B virus; MFI, mean fluorescence intensity; PD-L1, programmed death-ligand 1.

Table S4. Expression of restricted markers on patient HCC infiltrating NK cells

MFI / Groups	CD103	CD127	CD25	CD278	CD38	CD80	CD86	CTLA-4	GZMB	Ki-67	LAG-3	OX40	PD-1	TIGIT	TIM-3
No background HCC	0.00	0.00	0.00	0.00	40.40	0.00	0.00	0.00	28.66	0.51	0.00	0.00	0.00	1.54	0.00
AFL_HCC	0.00	0.01	0.00	0.00	19.91	0.00	0.00	0.00	35.12	0.75	0.00	0.00	0.00	2.64	0.10
NAFL_HCC	0.00	0.00	0.00	0.00	47.75	0.00	0.00	0.00	21.77	0.26	0.00	0.00	0.00	0.17	0.00
HBV_HCC	1.32	0.00	0.00	0.22	40.06	0.00	0.00	0.00	36.63	1.08	0.18	0.00	0.00	6.57	0.43
HBV_AFL_HCC	0.00	2.11	0.00	0.00	41.97	0.00	0.00	0.00	20.68	0.19	0.00	0.00	0.00	1.05	0.00
HBV_NAFL	0.0	0.0	0.0	0.1	40.	0.0	0.0	0.0	32.	0.	0.	0.0	0.	3.7	0.

_HCC_1	0	3	0	4	55	0	0	0	20	61	00	0	00	6	12
HBV_NAFL_HCC_2	0.75	0.00	0.00	0.00	36.98	0.00	0.00	0.00	24.01	0.92	0.00	0.00	0.00	4.54	0.00

Abbreviation: HCC, hepatocellular carcinoma; AFL, alcoholic fatty liver; NAFL, non-alcoholic fatty liver; HBV, hepatitis B virus; MFI, mean fluorescence intensity; CTLA-4, cytotoxic T lymphocyte-associated antigen-4; GZMB, granzyme B; LAG-3, lymphocyte-activation gene 3; PD-1, programmed cell death protein 1; TIGIT, T cell immunoreceptor with Ig and ITIM domains; TIM-3, T-cell immunoglobulin and mucin-domain containing-3.

Table S5. Expression of restricted markers on patient HCC infiltrating $CD4^+$ T cells

MFI / Groups	CD103	CD127	CD25	CD278	CD38	CD80	CD86	CTLA-4	GZMB	Ki-67	LAG-3	OX40	PD-1	TIGIT	TIM-3
No background HCC	0.00	34.20	3.81	5.04	0.76	0.00	0.00	0.00	23.37	2.22	0.00	0.13	1.24	0.62	0.00
AFL_HCC	0.00	27.20	1.89	2.06	7.05	0.00	0.00	0.00	20.55	2.66	0.00	0.00	0.00	0.12	0.00
NAFL_HCC	0.00	17.10	2.42	3.70	7.81	0.00	0.00	0.00	19.26	2.38	0.00	0.18	0.64	0.03	0.00
HBV_HCC	1.2	32.	2.9	1.9	19.	0.0	0.1	0.0	22.	4.	0.	0.1	0.	0.6	0.

	7	47	6	0	42	0	8	0	19	09	20	0	61	9	00
HBV_AFL_HCC	0.00	26.82	1.35	7.38	12.90	0.00	0.00	0.00	12.07	2.62	0.00	0.00	1.95	0.35	0.00
HBV_NAFL_HCC_1	0.00	39.76	0.36	3.41	3.37	0.00	0.00	0.00	18.07	2.49	0.00	0.03	1.32	0.19	0.00
HBV_NAFL_HCC_2	0.82	31.66	5.01	2.16	3.01	0.00	0.00	0.00	10.38	2.64	0.00	0.29	0.59	0.51	0.00

Abbreviation: HCC, hepatocellular carcinoma; AFL, alcoholic fatty liver; NAFL, non-alcoholic fatty liver; HBV, hepatitis B virus; MFI, mean fluorescence intensity; CTLA-4, cytotoxic T lymphocyte-associated antigen-4; GZMB, granzyme B; LAG-3, lymphocyte-activation gene 3; PD-1, programmed cell death protein 1; TIGIT, T cell immunoreceptor with Ig and ITIM domains; TIM-3, T-cell immunoglobulin and mucin-domain containing-3.

Table S6. Clinical information of patients with HCC and the expression of PD-1 on their tumor-infiltrating $CD8^+$ T cells

Sample	HBV infection	Pathology type	Fatty liver	T stage	N satge	HBsAg (IU/mL)	PD-1 on $CD8^+$ T
G001	Negative	Nodular	None	T1+2	N0	N/A	6.17
G002	Negative	Nodular	NAFL	T1+2	N1	N/A	6.24
G003	Negative	Diffuse	AFL	T1+2	N1	N/A	6.24
G004	Negative	Diffuse	AFL+NAFL	T1+2	N0	N/A	8.18

G005	Negative	Diffuse	NAFL	T1+2	N0	N/A	3.83
G006	Negative	Diffuse	AFL+NAFL	T1+2	N0	N/A	4.15
G007	Negative	Nodular	None	T1+2	N0	N/A	4.15
G008	Negative	Diffuse	None	T1+2	N0	N/A	4.18
G009	Negative	Nodular	None	T1+2	N0	N/A	4.22
G010	Negative	Diffuse	AFL	T1+2	N0	N/A	4.25
G011	Negative	Nodular	None	T1+2	N0	N/A	4.26
G012	Negative	Diffuse	AFL	T1+2	N0	N/A	4.3
G013	Negative	Nodular	AFL	T1+2	N0	N/A	4.3
G014	Negative	Nodular	NAFL	T1+2	N1	N/A	4.3
G015	Negative	Diffuse	AFL	T1+2	N0	N/A	4.35
G016	Negative	Nodular	NAFL	T1+2	N1	N/A	4.39
G017	Negative	Diffuse	AFL	T1+2	N0	N/A	4.4
G018	Negative	Nodular	NAFL	T1+2	N0	N/A	4.41
G019	Negative	Diffuse	AFL	T1+2	N0	N/A	4.41
G020	Negative	Nodular	NAFL	T1+2	N0	N/A	4.47
G021	Negative	Nodular	NAFL	T1+2	N0	N/A	4.48
G022	Negative	Nodular	NAFL	T1+2	N0	N/A	4.48
G023	Negative	Nodular	NAFL	T1+2	N0	N/A	4.53
G024	Negative	Nodular	NAFL	T1+2	N0	N/A	4.53
G025	Negative	Diffuse	NAFL	T1+2	N0	N/A	4.56
G026	Negative	Diffuse	NAFL	T1+2	N0	N/A	4.62

G027	Negative	Nodular	None	T1+2	N0	N/A	4.62
G028	Negative	Diffuse	None	T1+2	N0	N/A	4.62
G029	Negative	Nodular	None	T1+2	N0	N/A	4.63
G030	Negative	Diffuse	None	T1+2	N0	N/A	4.63
G031	Negative	Diffuse	None	T1+2	N0	N/A	4.66
G032	Negative	N/A	NAFL	T3+4	N1	N/A	4.68
G033	Negative	Nodular	None	T1+2	N0	N/A	4.72
G034	Negative	Diffuse	None	T1+2	N0	N/A	4.73
G035	Negative	Nodular	None	T1+2	N0	N/A	4.74
G036	Negative	Nodular	None	T1+2	N0	N/A	4.75
G037	Negative	Nodular	AFL	T1+2	N1	N/A	4.77
G038	Negative	Diffuse	None	T1+2	N0	N/A	4.83
G039	Negative	Massive	None	T3+4	N0	N/A	4.83
G040	Negative	Diffuse	AFL	T1+2	N1	N/A	4.85
G041	Negative	Diffuse	None	T1+2	N0	N/A	5.03
G042	Negative	Diffuse	None	T1+2	N0	N/A	5.05
G043	Negative	Diffuse	None	T1+2	N0	N/A	5.05
G044	Negative	Diffuse	None	T1+2	N0	N/A	5.08
G045	Negative	Diffuse	NAFL	T1+2	N0	N/A	5.1
G046	Negative	Diffuse	AFL+NAFL	T1+2	N0	N/A	5.1
G047	Negative	Massive	AFL+NAFL	T3+4	N0	N/A	5.15

G048	Negative	Diffuse	NAFL	T1+2	N0	N/A	5.16
G049	Negative	Diffuse	NAFL	T1+2	N0	N/A	5.17
G050	Negative	Diffuse	NAFL	T1+2	N0	N/A	5.19
G051	Negative	Diffuse	NAFL	T1+2	N0	N/A	5.35
G052	Negative	Diffuse	None	T1+2	N0	N/A	5.35
G053	Negative	Diffuse	None	T1+2	N0	N/A	5.4
G054	Negative	Massive	AFL	T3+4	N1	N/A	5.4
G055	Negative	Nodular	NAFL	T1+2	N0	N/A	5.41
G056	Negative	Nodular	NAFL	T1+2	N0	N/A	5.45
G057	Negative	Nodular	NAFL	T1+2	N0	N/A	5.47
G058	Negative	Nodular	AFL	T1+2	N0	N/A	5.47
G059	Negative	Diffuse	NAFL	T1+2	N0	N/A	5.48
G060	Negative	Nodular	AFL	T1+2	N0	N/A	5.48
G061	Negative	Diffuse	None	T1+2	N0	N/A	5.5
G062	Negative	Diffuse	None	T1+2	N0	N/A	5.53
G063	Negative	Nodular	AFL	T1+2	N1	N/A	5.6
G064	Negative	Diffuse	NAFL	T1+2	N0	N/A	5.62
G065	Negative	Diffuse	NAFL	T1+2	N0	N/A	5.63
G066	Negative	Massive	NAFL	T3+4	N0	N/A	5.65
G067	Negative	Nodular	AFL	T1+2	N0	N/A	6.64
G068	Negative	Nodular	AFL	T1+2	N0	N/A	6.65
G069	Negative	Nodular	AFL	T1+2	N0	N/A	6.79
G070	Negative	Nodular	AFL	T1+2	N0	N/A	8.13

G071	Negative	Nodular	AFL	T1+2	N0	N/A	8.14
G071	Positive	Nodular	NAFL	T1+2	N0	119	5.66
G072	Positive	Nodular	AFL+NAFL	T1+2	N0	55	5.68
G073	Positive	Nodular	AFL	T1+2	N0	77	5.68
G074	Positive	Nodular	NAFL	T1+2	N0	101	5.71
G075	Positive	Diffuse	NAFL	T1+2	N0	73	5.76
G076	Positive	Nodular	NAFL	T1+2	N0	116	5.82
G077	Positive	Nodular	NAFL	T1+2	N0	69	5.82
G078	Positive	Diffuse	NAFL	T1+2	N0	118	5.85
G079	Positive	Nodular	NAFL	T1+2	N0	135	5.87
G080	Positive	Nodular	NAFL	T1+2	N0	101	5.96
G081	Positive	Diffuse	AFL+NAFL	T1+2	N0	118	6.03
G082	Positive	Nodular	AFL	T1+2	N0	132	6.1
G083	Positive	Diffuse	AFL+NAFL	T1+2	N0	149	6.11
G084	Positive	Diffuse	AFL+NAFL	T1+2	N0	77	6.11
G085	Positive	Diffuse	AFL	T1+2	N0	80	6.13
G086	Positive	Diffuse	NAFL	T1+2	N0	23	3.45
G087	Positive	Nodular	NAFL	T1+2	N0	35	3.78
G088	Positive	Nodular	AFL	T1+2	N0	35	3.82

G089	Positive	Nodular	NAFL	T1+2	N1	92	6.25
G090	Positive	Diffuse	AFL	T1+2	N0	148	6.26
G091	Positive	Nodular	AFL	T1+2	N0	81	6.27
G092	Positive	Diffuse	AFL	T1+2	N0	122	6.29
G093	Positive	Nodular	AFL	T1+2	N0	122	6.48
G094	Positive	Diffuse	AFL	T1+2	N0	191	6.92
G095	Positive	Nodular	NAFL	T1+2	N0	127	6.96
G096	Positive	Diffuse	NAFL	T1+2	N0	158	6.98
G097	Positive	Diffuse	NAFL	T1+2	N0	140	7.12
G098	Positive	Nodular	NAFL	T1+2	N0	82	7.14
G099	Positive	Diffuse	NAFL	T1+2	N0	86	7.16
G100	Positive	Nodular	NAFL	T1+2	N0	162	7.17
G101	Positive	Nodular	NAFL	T1+2	N0	117	7.18
G102	Positive	Massive	None	T3+4	N0	138	7.18
G103	Positive	Nodular	AFL	T1+2	N0	121	7.22
G104	Positive	Massive	None	T3+4	N0	165	7.26
G105	Positive	Diffuse	None	T1+2	N0	80	7.28
G106	Positive	Nodular	AFL	T1+2	N0	119	7.29
G107	Positive	Nodular	None	T1+2	N0	154	7.57
G108	Positive	Nodular	AFL	T1+2	N1	179	7.65
G109	Positive	Nodular	None	T1+2	N0	194	7.66
G110	Positive	Diffuse	AFL	T1+2	N0	95	7.66
G111	Positive	Diffuse	None	T1+2	N0	184	7.8

G112	Positive	Diffuse	AFL	T1+2	N0	124	7.97
G113	Positive	Nodular	AFL+NAFL	T1+2	N0	143	8.05
G114	Positive	Diffuse	AFL+NAFL	T1+2	N0	127	8.09
G115	Positive	Diffuse	AFL	T1+2	N0	100	8.09
G116	Positive	Diffuse	AFL+NAFL	T1+2	N0	146	8.1
G117	Positive	Diffuse	AFL+NAFL	T1+2	N0	115	8.21
G118	Positive	Massive	NAFL	T3+4	N1	125	8.24
G119	Positive	Nodular	NAFL	T1+2	N1	156	8.29
G120	Positive	Nodular	NAFL	T1+2	N0	155	8.3
G121	Positive	Nodular	NAFL	T1+2	N0	127	8.33
G122	Positive	Diffuse	AFL	T1+2	N0	137	8.38
G123	Positive	Diffuse	None	T1+2	N0	200	8.43
G124	Positive	Massive	None	T3+4	N0	120	8.44
G125	Positive	Diffuse	AFL	T1+2	N0	193	8.49
G126	Positive	Nodular	None	T1+2	N0	195	8.64
G127	Positive	Nodular	None	T1+2	N0	196	8.65
G128	Positive	Nodular	None	T1+2	N0	169	8.78
G129	Positive	Diffuse	None	T1+2	N0	125	8.82
G130	Positive	Diffuse	None	T1+2	N0	182	9.11

G131	Positive	Nodular	None	T1+2	N0	152	9.2
G132	Positive	Nodular	None	T1+2	N0	202	9.28
G133	Positive	Diffuse	NAFL	T1+2	N0	193	9.36
G134	Positive	Nodular	NAFL	T1+2	N0	214	9.4
G135	Positive	Diffuse	NAFL	T1+2	N0	121	9.49
G136	Positive	Nodular	NAFL	T1+2	N0	196	9.51
G137	Positive	Diffuse	NAFL	T1+2	N0	154	9.63
G138	Positive	Nodular	AFL	T1+2	N0	150	9.64
G139	Positive	Nodular	AFL+NAFL	T1+2	N0	135	9.69
G140	Positive	Nodular	AFL+NAFL	T1+2	N0	173	9.72
G141	Positive	Nodular	AFL+NAFL	T1+2	N0	131	9.73
G142	Positive	Nodular	AFL+NAFL	T1+2	N0	204	9.82
G143	Positive	Nodular	None	T1+2	N0	161	10.1
G144	Positive	Nodular	None	T1+2	N0	161	10.3
G145	Positive	Nodular	AFL	T1+2	N1	129	10.5
G146	Positive	Nodular	None	T1+2	N0	215	10.5
G147	Positive	Nodular	None	T1+2	N0	193	10.5
G148	Positive	Nodular	None	T1+2	N0	225	10.7
G149	Positive	Nodular	AFL	T1+2	N0	159	10.8

G150	Positive	Nodular	AFL	T1+2	N0	202	10.9
G151	Positive	Nodular	AFL	T1+2	N0	198	11
G152	Positive	Nodular	AFL	T1+2	N0	207	11.2
G153	Positive	Diffuse	AFL	T1+2	N0	177	11.2
G154	Positive	Diffuse	None	T1+2	N0	209	11.2
G155	Positive	Nodular	None	T1+2	N0	214	11.5
G156	Positive	Nodular	None	T1+2	N0	155	11.6
G157	Positive	Nodular	None	T1+2	N0	150	12.5
G158	Positive	Diffuse	None	T1+2	N0	228	12.8
G159	Positive	Nodular	None	T1+2	N0	225	12.9
G160	Positive	Nodular	AFL	T1+2	N0	213	13
G161	Positive	Nodular	NAFL	T1+2	N0	184	14.8
G162	Positive	Nodular	NAFL	T1+2	N0	156	15.6
G163	Positive	Diffuse	AFL	T1+2	N0	157	15.9
G164	Positive	Diffuse	NAFL	T1+2	N0	179	18.3
G165	Positive	Diffuse	NAFL	T1+2	N0	71	6.8
G166	Positive	Diffuse	AFL	T1+2	N0	99	6.86
G167	Positive	Diffuse	None	T1+2	N0	40	5.23
G168	Positive	Diffuse	AFL+NAFL	T1+2	N0	63	5.25
G169	Positive	Massive	None	T3+4	N0	50	5.25
G170	Positive	Massive	None	T3+4	N1	87	5.26
G171	Positive	Nodular	AFL	T1+2	N1	65	5.28

G172	Positive	Diffuse	None	T1+2	N0	76	5.28
G173	Positive	Nodular	AFL	T1+2	N0	84	5.3
G174	Positive	Nodular	None	T1+2	N0	137	6.89
G175	Positive	Diffuse	None	T1+2	N0	99	6.91
G176	Positive	Diffuse	None	T1+2	N0	121	8.12
G177	Positive	Diffuse	AFL	T1+2	N1	24	4.93
G178	Positive	Nodular	None	T1+2	N0	38	4.96
G179	Positive	Nodular	None	T1+2	N0	50	4.97
G180	Positive	Nodular	None	T1+2	N0	56	4.99
G181	Positive	Nodular	None	T1+2	N0	75	5
G182	Positive	Nodular	None	T1+2	N0	48	5.01
G183	Positive	Nodular	AFL	T1+2	N1	34	3.28
G184	Positive	Diffuse	None	T1+2	N0	35	4.85
G185	Positive	Nodular	None	T1+2	N0	20	4.85
G186	Positive	Nodular	AFL	T1+2	N1	23	4.86
G187	Positive	Nodular	None	T1+2	N0	24	4.91
G188	Positive	Massive	None	T3+4	N0	51	5.03
G189	Positive	Massive	None	T3+4	N0	63	5.03

Abbreviation: HCC, hepatocellular carcinoma; AFL, alcoholic fatty liver; NAFL, non-alcoholic fatty liver; HBV, hepatitis B virus; HBsAg, Hepatitis B surface antigen; PD-1, programmed cell death protein 1.

Table S7. HBV-specific antigen or antibody content in blood of HBV-positive patients

Sample	HBsAg (IU/mL)	HBsAb (IU/mL)	HBeAg (S/CO)	HBeAb (S/CO)	HBcAb (S/CO)	Group
sample-018	0.5	18.92	0.39	2.05	1.33	HBsAg-low
sample-020	1.4	26.01	0.35	1.76	1.45	
sample-031	1.9	104.61	0.35	1	4.98	
sample-035	0.66	261.85	0.31	2.05	0.11	
sample-037	0.41	104.52	0.32	0.26	4.42	
sample-011	0.17	0.23	0.32	0.09	5.23	
sample-003	4.34	1.22	0.37	0.02	6.27	
sample-013	9.15	2.79	0.44	0.03	4.88	
sample-022	18.69	0.9	0.34	0.04	4.9	
sample-040	20.81	0.88	0.4	0.01	6.9	
sample-006	26.49	0.86	0.4	0.5	6.54	HBsAg-medium
sample-023	41.65	0.28	0.38	0.13	4.94	
sample-005	55.26	1.07	0.36	0.02	5.67	
sample-039	84	4.63	0.38	0.08	6.12	
sample-016	97.56	1.79	0.36	0.01	4.92	
sample-017	106.36	0.5	0.02	0.02	0	
sample-012	114.17	0.77	0.28	0.01	5.03	
sample-024	135.33	0.47	0.31	0.02	5.13	
sample-001	161.51	0.11	0.22	0.01	6.24	
sample-007	225.47	3.02	0.37	0.02	6.35	
sample-036	251	0	0.7	1.84	5.25	HBsAg-

sample-038	250	0.03	0.73	1.35	6.96	high
sample-025	349.34	3.29	1.19	1.85	4.56	
sample-010	402.84	0.5	0.46	0.02	5.89	
sample-029	938.56	33.53	11.11	1.44	5.71	
sample-034	1062.37	3.22	0.4	1.2	4.84	
sample-028	2196.43	0	0.36	0.01	5.41	
sample-008	250	0.22	0.34	0.02	5.41	
sample-009	250	3.27	0.45	0.04	4.8	
sample-004	3214.58	1.49	0.46	0.38	6.2	
sample-002	250	0.36	12.39	1.14	5.92	HBV-HCC
sample-032	461.26	0	0.41	0.72	5.2	
sample-030	489.04	0.41	0.35	0.01	5.25	
sample-021	629.23	0.89	0.36	0.01	5.01	
sample-026	641.4	0.21	0.83	1.54	5.16	
sample-014	250	0.21	0.37	0.02	5.08	
sample-015	250	0.5	0.37	0.02	4.65	
sample-033	250	0.26	0.4	0.01	5.6	
sample-027	3277.49	0	2.05	1.38	5.69	
sample-019	3417.11	9.9	0.92	0.63	5.47	

Abbreviation: HBV, hepatitis B virus; HBsAg, hepatitis B surface antigen; HBsAb, hepatitis B surface antibody; HBeAg, hepatitis B envelope antigen; HBeAb, hepatitis B envelope antibody; HBcAb, hepatitis B core antibody.

Table S8. HBV-specific antigen or antibody content in blood of HBV-Tg mice

	HBsAg	HBsAb	HBeAg	HBeAb	HBcAb
sample-001	4.82	10.17	68.5	2	1.96
sample-002	6.35	12.09	217.76	3.45	1.58
sample-003	3.15	11.86	42.58	1.97	1.91
sample-004	3.57	13.96	134.56	2.69	1.72
sample-005	0.05	26.19	22.05	1.4	1.4
sample-006	0.19	35.02	18.43	1.59	1.56
sample-007	18.16	25.48	68.24	3.27	1.1
sample-008	66.86	19.44	370.26	4.57	0.09
sample-009	169.95	29.75	736.81	4.75	0.05
sample-010	59.01	37.8	313.25	9.08	0.13
sample-011	63.7	34.51	383.38	6.9	0.16
sample-012	69.45	20.56	139.5	2.72	0.76

Abbreviation: HBV, hepatitis B virus; HBV-Tg, HBs-transgenic; HBsAg, hepatitis B surface antigen; HBsAb, hepatitis B surface antibody; HBeAg, hepatitis B envelope antigen; HBeAb, hepatitis B envelope antibody; HBcAb, hepatitis B core antibody.

Table S9. HBV-specific antigen or antibody content in blood of mice constructed with rAAV8-1.3HBV

Sample	HBsAg (IU/mL)	HBsAb (IU/mL)	HBeAg (S/CO)	HBeAb (S/CO)	HBcAb (S/CO)	Group

P16	0.1	5	0.2	18.1	20.9	HBsAg-low
P05	16	152.3	189.6	18.7	19.8	
P38	17.6	312	1000.8	28.2	15.7	
P10	17.9	123.6	724.3	23.6	19.5	
P33	22.5	320.1	870.5	29	15.7	
P03	30.7	124.6	417.6	20	19.4	
P15	110.5	174.1	1016.5	22.8	16.3	HBsAg-medium
P11	118.3	231.4	4215.6	66	9.6	
P13	124.1	226.2	4119.1	68.7	9.1	
P31	132.6	295.4	3906.3	65.8	7.2	
P32	132.6	295.4	3906.3	65.8	7.2	
P19	132.7	152.9	1536.2	28.4	14.8	
P26	643	276.1	3588.8	62.6	3.1	HBsAg-high
P35	644.9	344.6	3566	77.3	1.7	
P27	681.5	310.2	4147.9	55.7	2.4	
P42	751.3	197.3	2371.7	20.4	1.2	
P39	758.5	211.1	2472.4	22	1.2	
P29	760.9	236.1	3020.4	29.2	1.1	
P37	271.4	288.4	3994	70.2	3.9	HBV-HCC
P21	281.8	242.9	1150	24.9	3.9	
P40	768.2	204.2	2374.1	21.1	1.1	
P41	768.2	204.2	2374.1	21.1	1.1	
P30	796.7	233.8	3011.8	28.8	1.2	

P24	1001.3	215.8	3717.5	27.3	13.5	

Abbreviation: HBV, hepatitis B virus; rAAV8, recombinant Adeno-associated virus 8; HBsAg, hepatitis B surface antigen; HBsAb, hepatitis B surface antibody; HBeAg, hepatitis B envelope antigen; HBeAb, hepatitis B envelope antibody; HBcAb, hepatitis B core antibody.

SUPPLEMENTAL MATERIALS

The antibodies, chemical reagents, commercial kits, and data analysis software used in this study.

**REAGENT or RESOURCE**	**RESOURCE**	**IDENTIFIER**
**Antibodies**		
FITC anti-human CD45 Antibody	Biolegend	Cat# 304005
PE anti-human CD8	Biolegend	Cat# 980902
BV421 anti-human CD279 (PD-1) Antibody	Biolegend	Cat# 367421
FITC anti-human CD279 (PD-1) Antibody	Biolegend	Cat# 379205
FITC anti-mouse CD279 (PD-1) Antibody	Biolegend	Cat# 135213
PE anti-human CD152 (CTLA-4) Antibody	Biolegend	Cat# 369603
PE anti-mouse CD152	Biolegend	Cat# 106305

(CTLA-4) Antibody		
Brilliant Violet 421™ anti-human CD366 (Tim-3) Antibody	Biolegend	Cat# 345007
Brilliant Violet 421™ anti-mouse CD366 (Tim-3) Antibody	Biolegend	Cat# 134019
TOX Polyclonal Antibody	Life Technologies	Cat# PA5-34423
Brilliant Violet 421™ Donkey anti-rabbit IgG (minimal x-reactivity) Antibody	Biolegend	Cat# 406410
PerCP/Cyanine5.5 anti-human CD8a Antibody	Biolegend	Cat# 344709
PerCP/Cyanine5.5 anti-mouse CD8a Antibody	Biolegend	Cat# 100733
Brilliant Violet 605™ anti-human CD4 Antibody	Biolegend	Cat# 300555
Brilliant Violet 605™ anti-mouse CD4 Antibody	Biolegend	Cat# 100451
Hepatitis B Virus Surface Monoclonal Antibody	Life Technologies	Cat# MA1-7603
APC anti-mouse IgG1 Antibody	Biolegend	Cat# 406609

(89-Y) anti-human CD45, purified	Biolegend	Cat# 304001
(106-Cd) anti-human CD11c, purified	Biolegend	Cat# 337221
(110-Cd) anti-human CD19, purified	Biolegend	Cat# 302201
(112-Cd) anti-human CD62L, purified	Biolegend	Cat# 304835
(113-Cd) anti-human CD206, purified	Biolegend	Cat# 321127
(114-Cd) anti-human CD3, purified	Biolegend	Cat# 300401
(116-Cd) anti-human CD335, purified	Biolegend	Cat# 331902
(141-Pr) anti-human CD1c, purified	Biolegend	Cat# 331501
(142-Nd) anti-human CD40, purified	Biolegend	Cat# 334325
(143-Nd) anti-human CD278, purified	Biolegend	Cat# 313502
(144-Nd) anti-human CD38, purified	Biolegend	Cat# 303502
(145-Nd) anti-human CD163,	Biolegend	Cat# 333602

purified		
(146-Nd) anti-human CD8A, purified	Biolegend	Cat# 301053
(148-Nd) anti-human CD14, purified	Biolegend	Cat# 301801
(149-Sm) anti-human CD25, purified	Biolegend	Cat# 302602
(150-Nd) anti-human LAG3, purified	Biolegend	Cat# 369202
(151-Eu) anti-human CD103, purified	Biolegend	Cat# 350202
(152-Sm) anti-human CD204, purified	Biolegend	Cat# 371902
(153-Eu) anti-human TIGIT, purified	Biolegend	Cat# 372702
(154-Sm) anti-human TIM-3, purified	Biolegend	Cat# 345019
(155-Gd) anti-human PD-1, purified	Biolegend	Cat# 329941
(156-Gd) anti-human CD86, purified	Biolegend	Cat# 305435
(159-Tb) anti-human GITR, purified	Biolegend	Cat# 371202

(160-Gd) anti-human CD28, purified	Biolegend	Cat# 302937
(161-Dy) anti-human CTLA4, purified	Biolegend	Cat# 525401
(163-Dy) anti-human FOXP3, purified	Biolegend	Cat# 364702
(163-Dy) anti-human CD172a, purified	Biolegend	Cat# 372102
(164-Dy) anti-human FAS, purified	Biolegend	Cat# 305631
(166-Er) anti-human CD24, purified	Biolegend	Cat# 311127
(167-Er) anti-human CD27, purified	Biolegend	Cat# 302802
(168-Er) anti-human Ki-67, purified	Biolegend	Cat# 350523
(169-Tm) anti-human CD80, purified	Biolegend	Cat# 370610
(170-Er) anti-human HLA-DR, purified	Biolegend	Cat# 307602
(171-Yb) anti-human CCL22, purified	Biolegend	Cat# 694403
(172-Yb) anti-human	Biolegend	Cat# 341602

CX3CR1, purified		
(173-Yb) anti-human GZMB, purified	Biolegend	Cat# 372202
(174-Yb) anti-human CD4, purified, purified	Biolegend	Cat# 300501
(175-Lu) anti-human CD274, purified, purified	Biolegend	Cat# 329719
(209Bi) anti-human CD16, purified	Biolegend	Cat# 302051
(89-Y) anti-mouse CD45, purified	Biolegend	Cat# 103101
(115-In) anti-mouse CD3e, purified	Biolegend	Cat# 152302
(142-Nd) anti-mouse CD127, purified	Biolegend	Cat# 135002
(143-Nd) anti-mouse TIM-3, purified	Biolegend	Cat# 165402
(144-Nd) anti-mouse CD44, purified	Biolegend	Cat# 156002
(145-Nd) anti-mouse CTLA-4, purified	Biolegend	Cat# 106202
(150-Nd) anti-mouse CD25, purified	Biolegend	Cat# 101902

(151-Eu) anti-mouse GZMB, purified	Biolegend	Cat# 396402
(152-Sm) anti-mouse CD19, purified	Biolegend	Cat# 115502
(153-Eu) anti-mouse CD62L, purified	Biolegend	Cat# 104402
(157-Gd) anti-mouse CD335, purified	Biolegend	Cat# 137625
(160-Gd) anti-mouse CD45R, purified	Biolegend	Cat# 103201
(162-Dy) anti-mouse FOXP3, purified	Biolegend	Cat# 320001
(167-Er) anti-mouse F4/80, purified	Biolegend	Cat# 123143
(171-Yb) anti-mouse CD69, purified	Biolegend	Cat# 104533
(172-Yb) anti-mouse PD-1, purified	Biolegend	Cat# 135202
(173-Yb) anti-mouse ICOS, purified	Biolegend	Cat# 313502
(158-Gd) anti-mouse PD-L1, purified	Biolegend	Cat# 124301
(197-Au) anti-mouse CD4,	Biolegend	Cat# 100505

purified		
(198-Pt) anti-mouse CD8a, purified	Biolegend	Cat# 100755
(209-Bi) anti-mouse CD11b, purified	Biolegend	Cat# 101201
(165-Ho) anti-mouse TCRgd, purified	Biolegend	Cat# 107515
(164-Dy) anti-mouse TCF-7, purified	Biolegend	Cat# 655202
(156-Gd) anti-mouse TOX, purified	Biolegend	Cat# 682601
(141-Pr) anti-mouse CD163, purified	Biolegend	Cat# 156702
(155-Gd) anti-mouse CD80, purified	Biolegend	Cat# 104735
anti-CD44 antibody	Abcam	Cat# ab243894
anti-GZMB antibody	Abcam	Cat# ab255598
anti-PD1 antibody	Abcam	Cat# ab52587
anti-TIM-3 antibody	Abcam	Cat# ab241332
Goat Anti-Mouse IgG H&L (FITC)	Abcam	Cat# ab6785
Goat Anti-Rabbit IgG H&L (Cy5®)	Abcam	Cat# ab6564

**Chemicals, peptides, and recombinant proteins**		
rAAV8-1.3HBV	Beijing FivePlus Gene Technology Co., Ltd.	Cat# AMV-002
16% paraformaldehyde aqueous solution	Electron Microscopy Sciences/ LucernaChem	Cat# 15710
2-methylbutane	Sigma Aldrich	Cat# M32631
Bovine Serum Albumin (BSA)	Sigma-Aldrich	Cat# B2064
Fetal bovine serum (FBS)	ThermoFisher Scientific	Cat# 12484028
Collagenase from Clostridium histolyticum, type IV (Collagenase IV)	Sigma-Aldrich	Cat# C5138
Deoxyribonuclease I from bovine pancreas (DNase I)	Sigma-Aldrich	Cat# DN25-1G
Dimethyl sulfoxide (DMSO)	Fischer Bioreagents	Cat# BP231-1
Ethylenediaminetetraacetic acid (EDTA)	StemCell Technologies, Inc	Cat# EDS-100G
Diethylnitrosamine (DEN)	Sigma-Aldrich	Cat# N0258
Carbon tetrachloride (CCl_4)	Sigma-Aldrich	Cat# 488488
**Critical commercial assays**		
Hematoxylin-Eosin (HE) Stain Kit	Solarbio	Cat# G1120

DAB Horseradish Peroxidase Color Development Kit	Beyotime	Cat# P0202
LS Column Adapter	Miltenyi	Cat# 130-090-544
EQ Four Element Calibration	Fluidigm	Cat# 201078
Cell Staining Buffer	Biolegend	Cat# 420201
Antibody Stabilizer	Candor Bioscience	Cat# 131050
Antifading Mounting Medium with DAPI	Dianova	Cat# SCR-038448
Triton X-100	Solarbio	Cat# T8200
Sudan black B	MedChemExpress	Cat# HY-D0213
Hanks' Balanced Salt Solution (HBSS)	Beyotime	Cat# C0219
Ethanol	Sigma	Cat# 493511
Bambanker	LubioScience GmbH	Cat# 523303 (BB02)
Cell-ID™ Intercalator-Ir	Fluidigm	Cat# 201192B
Cell-ID Cisplatin	Fluidigm	Cat# 201064
Cryo Embedding Medium	Medite	Cat# 41-3011-00
Dead Cell Removal Kit	Miltenyi	Cat# 30-090-101
Human TruStain FcX™ (Fc Receptor Blocking Solution)	Biolegend	Cat# 422302
TruStain FcX™ (anti-mouse CD16/32) Antibody	Biolegend	Cat# 101319
FOXP3 Fix/Perm Buffer Set	Biolegend	Cat# 421403

Indium (^{115}-In)	Trace Sciences International	N/A
Iridium (^{191}-Ir, ^{193}-Ir)	Fluidigm	Cat# 201192A
Isoflurane	Minrad	N/A
Maxpar X8 Multimetal Labeling Kit	Fluidigm	Cat# 201300
Maxpar Fix and Perm Buffer	Fluidigm	Cat# 201067
Percoll	Sigma-Aldrich	Cat# GE17-0891
Phosphate-buffered saline (PBS)	Life Technologies	Cat# 14190094
Roswell Park Memorial Institute (RPMI) 1640 Medium	Life Technologies	Cat# 42401042
InVivoMAb anti-mouse PD-1 (CD279)	Bioxcell	Cat# BE0146
Mouse ALT ELISA Kit	Abcam	Cat# ab282882
Mouse AST ELISA Kit	Abcam	Cat# ab263882
Gamma Glutamyl Transferase (GGT) Assay Kit (Colorimetric)	Abcam	Cat# ab241029
Mouse ALP ELISA Kit	Abcam	Cat# ab267583
HBsAg Quantitative Kit	Abbott	N/A
HBsAb Quantitative Kit	Abbott	N/A
HBeAg Quantitative Kit	Abbott	N/A

HBeAb Quantitative Kit	Abbott	N/A
HBcAb Quantitative Kit	Abbott	N/A
Lieber-DeCarli	Trophic Animal Feed High-Tech Co. Ltd (Nanjing, China)	N/A
Clinton-Cybulsky	Trophic Animal Feed High-Tech Co. Ltd (Nanjing, China)	N/A
**Software and algorithms**		
GraphPad Prism (version 8.0)	GraphPad Software Inc	https://www.graphpad.com/scientificsoftware/prism/
FlowJo (version7.6.1)	FlowJo, LLC	https://www.flowjo.com/solutions/flowjo
Cytobank	Beckman, LLC	https://premium.cytobank.cn/cytobank/experiments
MATLAB R2016a	N/A	https://www.mathworks.com/
Normalizer	(1)	https://github.com/nolanlab/bead-normalization/releases
R version 3.5	R Development Core Team	https://www.r-project.org/
R Studio	RStudio Team, 2015	https://www.rstudio.com/
FlowSOM	(2)	https://github.com/SofieVG/FlowSOM
Circlize	(3)	https://cran.r-project.org/web/

		packages/circlize/index.html
MCF-data-analysis	(4)	https://github.com/hartmannfj/ MCF-data-analysis
CyTOF workflow	(5)	https://f1000research.com/articles/6-748#
t-SNE	(6)	https://github.com/jkrijthe/Rtsne
One-SENSE	(7)	N/A
flowStats	N/A	https://www.bioconductor.org/ packages/release/bioc/html/flowStats.html
pheatmap	N/A	https://cran.r-project.org/web/packages/pheatmap/index.html
TCGAbiolinks	(8)	https://bioconductor.org/packages/release/bioc/html/TCGAbiolinks.html
flowCore	(9)	N/A
flowWorkspaceData	(10)	N/A
Hmisc	N/A	https://cran.r-project.org/web/packages/Hmisc/index.html
ggplot2	N/A	https://cran.r-project.org/web/

		packages/ggplot2/index.html

Supplemental References

1. Finck R, Simonds EF, Jager A, Krishnaswamy S, Sachs K, Fantl W, Pe'er D, Nolan GP, Bendall SC. 2013. Normalization of mass cytometry data with bead standards. Cytometry A 83:483-94.
2. Van Gassen S, Callebaut B, Van Helden MJ, Lambrecht BN, Demeester P, Dhaene T, Saeys Y. 2015. FlowSOM: Using self-organizing maps for visualization and interpretation of cytometry data. Cytometry A 87:636-45.
3. Gu Z, Gu L, Eils R, Schlesner M, Brors B. 2014. circlize Implements and enhances circular visualization in R. Bioinformatics 30:2811-2.
4. Hartmann FJ, Bernard-Valnet R, Quériault C, Mrdjen D, Weber LM, Galli E, Krieg C, Robinson MD, Nguyen XH, Dauvilliers Y, Liblau RS, Becher B. 2016. High-dimensional single-cell analysis reveals the immune signature of narcolepsy. J Exp Med 213:2621-2633.
5. Nowicka M, Krieg C, Crowell HL, Weber LM, Hartmann FJ, Guglietta S, Becher B, Levesque MP, Robinson MD. 2017. CyTOF workflow: differential discovery in high-throughput high-dimensional cytometry datasets. F1000Res 6:748.
6. Melit Devassy B, George S, Nussbaum P. 2020. Unsupervised Clustering of Hyperspectral Paper Data Using t-SNE. J Imaging 6.
7. Cheng Y, Wong MT, van der Maaten L, Newell EW. 2016. Categorical Analysis of Human T Cell Heterogeneity with One-Dimensional Soli-Expression by Nonlinear Stochastic Embedding. J Immunol 196:924-32.
8. Colaprico A, Silva TC, Olsen C, Garofano L, Cava C, Garolini D, Sabedot TS, Malta TM, Pagnotta SM, Castiglioni I, Ceccarelli M, Bontempi G, Noushmehr H. 2016. TCGAbiolinks: an R/Bioconductor package for integrative analysis of TCGA data. Nucleic Acids Res 44:e71.
9. Hahne F, LeMeur N, Brinkman RR, Ellis B, Haaland P, Sarkar D, Spidlen J, Strain E, Gentleman R. 2009. flowCore: a Bioconductor package for high throughput flow cytometry. BMC Bioinformatics 10:106.
10. Finak G, Jiang W, Pardo J, Asare A, Gottardo R. 2012. QUAliFiER: an automated pipeline for quality assessment of gated flow cytometry data. BMC Bioinformatics 13:252.
